# Co-translational biogenesis of lipid droplet integral membrane proteins

**DOI:** 10.1242/jcs.259220

**Published:** 2021-11-02

**Authors:** Pawel Leznicki, Hayden O. Schneider, Jada V. Harvey, Wei Q. Shi, Stephen High

**Affiliations:** 1 School of Biological Sciences, Faculty of Biology, Medicine and Health, University of Manchester, Manchester, M13 9PT, UK; 2 Department of Chemistry, Ball State University, Muncie, IN 47306, USA

**Keywords:** Co-translational, Endoplasmic reticulum, Lipid droplets, Membrane proteins, Protein targeting

## Abstract

Membrane proteins destined for lipid droplets (LDs), a major intracellular storage site for neutral lipids, are inserted into the endoplasmic reticulum (ER) and then trafficked to LDs where they reside in a hairpin loop conformation. Here, we show that LD membrane proteins can be delivered to the ER either co- or post-translationally and that their membrane-embedded region specifies pathway selection. The co-translational route for LD membrane protein biogenesis is insensitive to a small molecule inhibitor of the Sec61 translocon, Ipomoeassin F, and instead relies on the ER membrane protein complex (EMC) for membrane insertion. This route may even result in a transient exposure of the short N termini of some LD membrane proteins to the ER lumen, followed by putative topological rearrangements that would enable their transmembrane segment to form a hairpin loop and N termini to face the cytosol. Our study reveals an unexpected complexity to LD membrane protein biogenesis and identifies a role for the EMC during their co-translational insertion into the ER.

## Abstract (precis)

**Summary:** Insertion of many lipid droplet membrane proteins into the ER is co-translational, mediated by the ER membrane protein complex and may involve topology reorientation.

## INTRODUCTION

Lipid droplets (LDs) are highly conserved dynamic organelles that are present in virtually all eukaryotic cells. They act as the main intracellular storage site for neutral lipids that can be used for energy production and membrane biosynthesis. LDs also regulate protein homeostasis (Cermelli et al., 2006; Moldavski et al., 2015) and play a key role in the propagation of viruses, including hepatitis C (Miyanari et al., 2007; Samsa et al., 2009; Zhang et al., 2018). The massive accumulation of LDs observed in pathological conditions, such as obesity, fatty liver disease (steatosis), diabetes and atherosclerosis, underscores their medical relevance, and mutations in LD proteins are linked to lipodystrophies and motor neuron diseases (Fujimoto and Parton, 2011; Henne et al., 2018; Krahmer et al., 2013).

LD formation is initiated when excess neutral lipids are deposited between the leaflets of the endoplasmic reticulum (ER) bilayer. A growing LD is then formed at discrete ER sites enriched in LD biogenesis factors, such as seipin and LDAF1 (Chung et al., 2019). The nascent LD subsequently buds off from the ER into the cytosol (Fujimoto and Parton, 2011; Henne et al., 2018; Krahmer et al., 2013), although there is compelling evidence that LDs can stay connected to the ER via membrane bridges (Wilfling et al., 2014, 2013; Zehmer et al., 2009). Hence, LD biogenesis at the ER results in the formation of a hydrophobic oil phase surrounded by a phospholipid monolayer derived from the cytosolic leaflet of the ER. Such an architecture distinguishes LDs from other intracellular membrane-enveloped organelles, which are surrounded by phospholipid bilayers, and has profound consequences for phospholipid packing and protein localisation to LDs (Caillon et al., 2020; Dhiman et al., 2020; Kory et al., 2015; Krahmer et al., 2011; Olarte et al., 2020).

Soluble proteins target LDs from the cytosol, using amphipathic helices, lipid anchors and protein-protein interactions to associate with them. Their binding is defined by protein crowding and phospholipid packing defects within the LD-surrounding monolayer (Chorlay and Thiam, 2020; Dhiman et al., 2020; Kory et al., 2015). In contrast, integral membrane proteins must first be inserted into the ER membrane prior to their trafficking to LDs (Ingelmo-Torres et al., 2009; Ostermeyer et al., 2004; Schrul and Kopito, 2016; Stevanovic and Thiele, 2013; Turro et al., 2006; Zehmer et al., 2009, 2008). Such integral membrane proteins include a number of lipid synthesising and metabolising enzymes that relocate from the ER to LDs to support LD growth (Wilfling et al., 2013), limit their lipolysis (Olzmann et al., 2013; Zhang et al., 2017) and potentially link LDs to protein quality control machinery (Klemm et al., 2011; Spandl et al., 2011). The energetic barrier associated with accommodating hydrophilic residues within the very hydrophobic LD interior, combined with the reduced thickness of the LD phospholipid monolayer as compared to phospholipid bilayers, mean that LD integral membrane proteins adopt a hairpin or amphipathic helix conformation in which both their N and C termini face the cytosol (Caillon et al., 2020; Olarte et al., 2020; Thiam et al., 2013). Indeed, fully membrane spanning proteins are specifically excluded from LDs (Khaddaj et al., 2022).

The majority of integral membrane proteins destined for the secretory pathway are co-translationally targeted to, and inserted into, the ER membrane via a pathway that is initiated by the signal recognition particle (SRP) (O'Keefe et al., 2021a). Hence, the SRP binds to a hydrophobic N-terminal signal sequence or the first transmembrane domain (TMD) of a nascent membrane protein as soon as it emerges from the ribosome, and targets the ribosome-nascent chain complex to the ER where the SRP interacts with its membrane-tethered receptor SR (a heterodimer of SRPRA, which is referred to here as SRα, and SRPRB). In most cases, the ribosome-nascent chain complex is then transferrsed to the Sec61 translocon, composed of Sec61α, Sec61β and Sec61γ, which opens laterally into the ER membrane to enable the release of newly synthesised TMD(s) into the bilayer (O'Keefe et al., 2021a). One hallmark of this Sec61-dependent pathway is its sensitivity to small molecules, including Ipomoeassin F, which strongly inhibit membrane protein integration at the ER (Luesch and Paavilainen, 2020; McKenna et al., 2017; O'Keefe et al., 2021b; Zong et al., 2019). Indeed, the insensitivity of so-called type III membrane proteins, defined by the lack of an N-terminal cleavable signal sequence and translocation of the N terminus to the ER lumen, to these small molecule inhibitors (McKenna et al., 2017; Zong et al., 2019) led to the identification of an alternative pathway for their co-translational integration into the ER membrane (O'Keefe et al., 2021c). This alternative pathway for the co-translational integration of type III membrane proteins relies on the ER membrane complex (EMC) (O'Keefe et al., 2021c), consistent with growing evidence that it can act as a standalone membrane insertase for certain types of TMDs (Bai et al., 2020; Chitwood et al., 2018; Guna et al., 2018; O'Donnell et al., 2020; Pleiner et al., 2020).

Relatively little is known about the biogenesis of LD membrane proteins at the ER. Studies of oleosins, major components of plant LDs, established that, at least in heterologous plant/mammalian and plant/yeast systems (Abell et al., 2002; Beaudoin et al., 2000), these proteins are targeted to the ER co-translationally via the action of SRP and SR. However, oleosins are characterised by a very hydrophobic membrane-embedded region of ∼72 amino acids (Murphy, 1993), which is substantially longer than the ∼30-amino-acid hairpin found in most LD membrane proteins, and hence the generality of these findings has been questioned (Dhiman et al., 2020). More recently, Schrul and Kopito (2016) carried out an elegant analysis of the ER membrane insertion requirements for a model LD membrane protein, UBXD8 (also known as FAF2). They found that UBXD8 is post-translationally inserted into the ER membrane with a preformed hairpin loop topology via a process that is mediated by soluble Pex19 and its membrane-tethered receptor Pex3 (Schrul and Kopito, 2016). Likewise, two reticulon-homology domain containing proteins, Arl6IP1 and Rtn4C, that reside in the ER membrane in a hairpin loop conformation are also suggested to follow this post-translational pathway (Yamamoto and Sakisaka, 2018). However, the biogenesis of other LD integral membrane proteins has not been studied in detail, leaving open the possibility of alternative biosynthetic pathways, as previously identified for tail-anchored membrane proteins (Casson et al., 2017).

To better understand their biogenesis at the ER, we have used a panel of well-defined LD membrane proteins and investigated the requirements for their delivery to, and insertion into, the ER membrane. We find that LD membrane proteins rely on at least two distinct biosynthetic pathways that are selected via the hydrophobic membrane-inserting region of individual LD proteins. One is a post-translational route that is used by UBXD8 and HSD17B7, and appears equivalent to the previously defined Pex19/Pex3-mediated pathway (Schrul and Kopito, 2016). The other is a co-translational route that is favoured by the majority of the LD membrane proteins we have tested. This co-translational pathway is mediated by the EMC and involves a transient exposure of the short N termini of LD membrane proteins to the ER lumen prior to their assembly into nascent LDs.

## RESULTS

### LD membrane proteins differ in their requirements for insertion into the ER

**Fig. 1.**
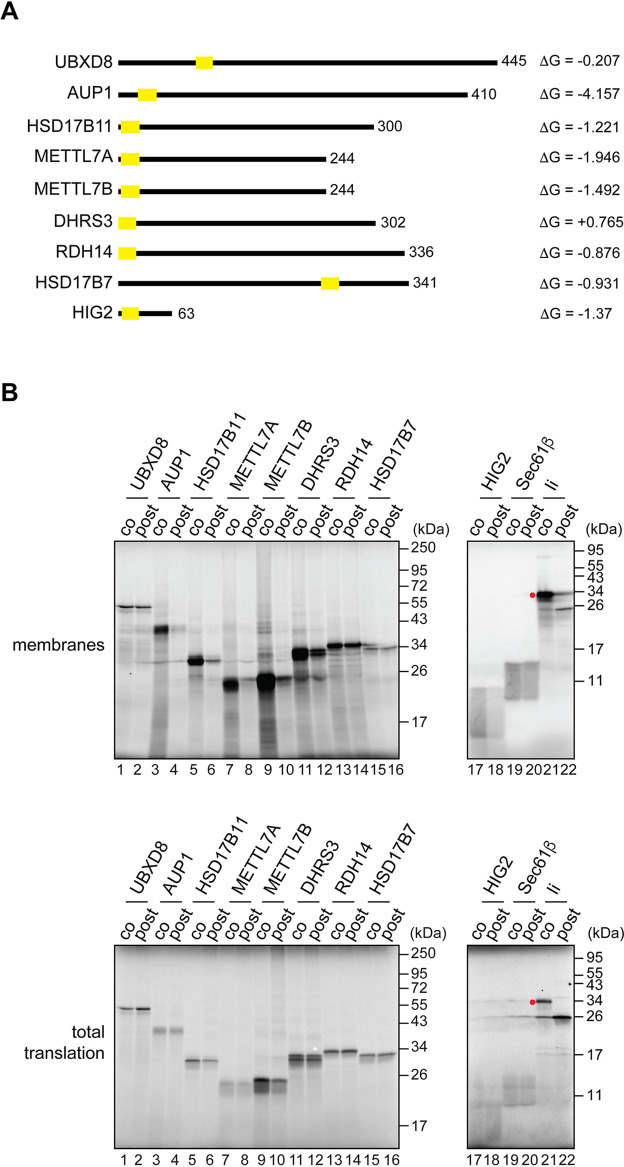
**LD membrane proteins differ in their requirements for delivery to the ER.** (A) A schematic representation of the LD membrane proteins used. Protein length, localisation of predicted TMDs (yellow rectangles) (ΔG predictor, Hessa et al., 2007) and estimated ΔG (in kcal/mol) associated with ER membrane insertion (Hessa et al., 2007) are indicated. (B) Untagged membrane proteins as indicated were synthesised *in vitro* using rabbit reticulocyte lysate and ER-derived microsomes, which were either present throughout the reaction (co-translational conditions; ‘co’) or added following translation termination (post-translational conditions; ‘post’). Membrane-associated material (top panels) and total translation reactions (bottom panels) were resolved by SDS-PAGE, and products were visualised by phosphorimaging. Sec61β and invariant chain (Ii; also known as HLA class II histocompatibility antigen gamma chain/CD74) are control proteins inserted into the ER membrane either post- or co-translationally, respectively. Red dot indicates the N-glycosylated species of Ii.

To investigate whether LD membrane proteins share a common pathway for their biogenesis at the ER, we compared the ER membrane insertion requirements of the well-studied UBXD8 with those of other known LD membrane proteins (Fig. 1A). Initially, we adopted the homologous *in vitro* translation system that was instrumental in identifying the Pex19/Pex3-dependent route for UBXD8 biogenesis (Schrul and Kopito, 2016). Furthermore, by carrying out LD membrane protein synthesis either in the presence of ER-derived membrane (co-translationally), or by adding the ER membrane following translation termination (post-translationally), we were able to establish the favoured mode of membrane binding/insertion for each LD protein studied (McKenna et al., 2016; Schrul and Kopito, 2016).

As previously reported (Schrul and Kopito, 2016), we found that UBXD8 bound to ER-derived microsomes equally well under co- and post-translational conditions akin to Sec61β, a member of the so-called tail-anchored membrane proteins that are delivered to the ER post-translationally (O'Keefe et al., 2021a) (Fig. 1B, lanes 1 and 2, and 19 and 20). Furthermore, this behaviour was mirrored at a qualitative level by two other LD membrane proteins, RDH14 and HSD17B7 (Fig. 1B, lanes 13 and 14, and 15 and 16). However, in contrast to these three examples, the membrane association of the majority of the other LD proteins analysed was dramatically reduced under conditions that required post-translational targeting to the ER (Fig. 1B, lanes 3-10, and 17 and 18), despite comparable levels of protein synthesis (Fig. 1B, bottom panel). Hence, these LD proteins behaved much like the invariant chain [Ii, also known as human leukocyte antigen (HLA) class II histocompatibility antigen gamma chain/CD74] (Fig. 1B, compare lanes 21 and 22), a well-studied substrate for the Sec61-mediated co-translational pathway of membrane insertion into the ER (Lipp and Dobberstein, 1986; Zong et al., 2020, 2019). In the case of DHRS3, we observed an intermediate effect, as evidenced by a modest reduction in its membrane association under post-translational conditions (Fig. 1B, compare lanes 11 and 12). We note that the TMD of DHRS3 appears less hydrophobic than any of the other LD membrane proteins studied (see Fig. 1A) and hence our data are consistent with the possibility that DHRS3 associates with the ER via an amphipathic helix rather than a fully membrane-inserted hairpin loop (Pataki et al., 2018).

Taken together, we conclude that LD membrane proteins differ in their capacity to be post-translationally inserted into the ER. Unlike UBXD8, it may be that other LD proteins are not effectively maintained in a membrane insertion competent form by cytosolic chaperones, such as Pex19 (Schrul and Kopito, 2016), or, alternatively, they employ a co-translational pathway for membrane insertion.

### LD membrane proteins expose their N termini to the ER lumen

Although the TMD of UBXD8 has been proposed to insert post-translationally into the ER membrane as a hairpin loop (Schrul and Kopito, 2016), the majority of membrane proteins that are co-translationally inserted into the ER expose hydrophilic regions that flank their TMD to the ER lumen (O'Keefe et al., 2021a). Given our finding that LD membrane proteins may not follow a single biosynthetic pathway, we wondered whether some LD membrane proteins may also translocate their short hydrophilic N terminus into the ER lumen during biogenesis. To address this question, we tagged our panel of LD membrane proteins (Fig. 1A) with a short N-terminal extension derived from bovine rhodopsin (OPG2), which contains two N-glycosylation sites (Leznicki et al., 2010; McKenna et al., 2016; O'Keefe et al., 2021b,c; Pedrazzini et al., 2000; Schrul and Kopito, 2016), and with a C-terminal FLAG epitope. As N-glycosylation is an ER lumen-specific modification, the presence of N-glycan(s) on the OPG2 tag can be used as a readout for its translocation to the ER lumen (Leznicki et al., 2010; McKenna et al., 2016; O'Keefe et al., 2021b,c; Pedrazzini et al., 2000; Schrul and Kopito, 2016).

**Fig. 2.**
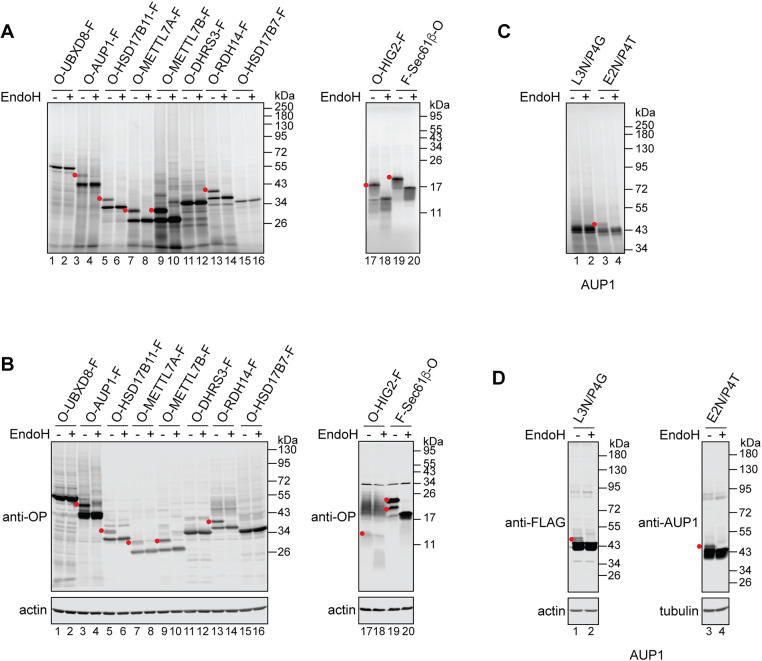
**LD membrane proteins translocate their N termini to the ER lumen.** (A) Indicated LD membrane proteins bearing an N-terminal rhodopsin-derived (OPG2) tag (‘O’), which contains two N-glycosylation sites, and a C-terminal FLAG epitope (‘F’) were translated *in vitro* in the presence of ER-derived microsomes. Membranes were isolated, N-glycosylated species (red dots) identified based on their altered mobility in SDS-PAGE following EndoH digestion, and products were visualised by phosphorimaging. Sec61β with an N-terminal FLAG and a C-terminal OPG2 tag was used as an ER-resident control protein that fully spans the lipid bilayer. (B) Proteins used in A were transiently expressed in U2OS cells, which were then lysed and, where indicated, treated with EndoH. Samples were resolved by SDS-PAGE and results visualised by western blotting using anti-rhodopsin (OP) and anti-actin antibodies. (C,D) Indicated variants of AUP1 with (AUP1^L3N/P4G^) or without (AUP1^E2N/P4T^) a C-terminal FLAG epitope were translated *in vitro* in the presence of ER-derived microsomes (C) or transiently expressed in U2OS cells (D), and their N-glycosylation status was tested by means of EndoH sensitivity. Results were visualised either by phosphorimaging (C) or by western blotting using the indicated antibodies (D).

Strikingly, when these OPG2-tagged LD membrane proteins were synthesised *in vitro* in the presence of ER-derived microsomes most of them migrated as two distinct species (see Fig. 2A for membrane fraction, and Fig. S1 for total translation products). By means of their sensitivity to endoglycosidase H (EndoH), we established that in each case the higher molecular weight species were N-glycosylated (Fig. 2A; Fig. S1, red dots) and hence their OPG2 tag had entered the ER lumen. The N-terminal location of the predicted TMD for most of the proteins analysed (see Fig. 1A) means that the distance between the N-glycosylation sites of the OPG2 tag and the active site of the oligosaccharyltransferase complex is relatively short (Nilsson and von Heijne, 1993). Hence, only the distal N-glycosylation site of the OPG2 tag is modified (Nilsson and von Heijne, 1993). Only three of the OPG2-tagged LD proteins showed no evidence of N-glycosylation: UBXD8, DHRS3 and HSD17B7 (Fig. 2A, lanes 1 and 2, 11 and 12, and 15 and 16), consistent with previous studies of UBXD8 (Schrul and Kopito, 2016) and DHRS3 (Pataki et al., 2018). In short, we find that the majority of LD proteins that preferentially associate with the ER membrane under co-translational conditions (Fig. 1B), can also be N-glycosylated when tagged at their N terminus with an OPG2 extension (Fig. 2A). This behaviour supports a model in which this group of LD membrane proteins share a common, most likely co-translational, pathway for their biogenesis at the ER. Importantly, the OPG2 and FLAG tags did not alter the biosynthetic pathway selection of the LD proteins tested (Fig. S2), and did not affect their delivery to LDs in oleic acid-loaded U2OS cells (Fig. S3).

To test our *in vitro* findings in a cell-based system, we transiently expressed the same tagged LD membrane proteins in U2OS cells and used EndoH sensitivity to test their N-glycosylation (Fig. 2B). We obtained qualitatively similar results to the *in vitro* assay (Fig. 2A,B), supporting the physiological significance of our findings. As the N terminus of neither UBXD8 nor HSD17B7 reaches the ER lumen (Fig. 2A,B, compare lanes 1 and 2, and 15 and 16) we wondered whether they may assume an opposite topology and translocate their C termini across the ER membrane. To address this question, we reversed the localisation of the OPG2 and FLAG epitopes. As we could not detect any N-glycosylation of the OPG2 tag when placed at either the N or C terminus of these two proteins, either *in vitro* (Fig. S4A) or in cultured cells (Fig. S4B), we conclude that UBXD8 and HSD17B7 follow a distinct biosynthetic pathway from the majority of LD membrane proteins we have tested. This pathway is characterised by its efficient operation under post-translational conditions and the lack of access to the ER lumen that it provides LD proteins at the ER membrane (Fig. 1B, Fig. 2A,B; Fig. S4). Only in the case of RDH14 did we observe comparable levels of membrane association under co- and post-translational conditions, and the efficient N-glycosylation of its N-terminal OPG2 tag (Fig. 1B, Fig. 2A,B). We conclude that RDH14 can likely access both co- and post-translational routes for LD membrane protein biogenesis at the ER (see Discussion).

Finally, to test whether we can detect LD membrane protein exposure to the ER lumen in the absence of an artificial OPG2 tag, we took advantage of the fact that the predicted TMD of AUP1 results in a slightly longer N-terminal region than in our other ‘co-translational’ LD membrane proteins (see Fig. 1A). This allowed us to create two AUP1 variants, AUP1^L3N/P4G^ and AUP1^E2N/P4T^, that bear single N-glycosylation sites engineered into their otherwise native sequence. We found that a fraction of AUP1^E2N/P4T^ was N-glycosylated both *in vitro* (Fig. 2C, lanes 3 and 4; Fig. S1) and in cells (Fig. 2D, lanes 3 and 4), whereas AUP1^L3N/P4G^ was N-glycosylated in cells but not *in vitro* (Fig. 2C,D, lanes 1 and 2; Fig. S1). Interestingly, a previous study of AUP1 (Stevanovic and Thiele, 2013) failed to detect N-glycosylation of the AUP1^L3N/P4G^ variant in COS-7 cells, indicating that modification of this particular engineered site may be sensitive to the experimental system used, as further evidenced by the difference between the *in vitro* and cell-based assays we observed with this variant. Taken together, our results strongly suggest that during their biogenesis at the ER a number of LD membrane proteins can translocate their short N-terminal hydrophilic domain into the ER lumen.

### Co-translationally inserted LD membrane proteins access the ER lumen transiently before being trafficked to LDs

The N-glycosylation of LD membrane proteins that we detected was typically incomplete (Fig. 2), raising the possibility that LD membrane proteins with their N termini translocated into the ER lumen may represent a distinct population of polypeptides that stably reside in the ER. As N-glycosylation occurs specifically in the ER lumen we reasoned that the presence of an N-glycosylated LD membrane protein on LDs would confirm that, having accessed the ER lumen, such proteins remain competent for sorting into LDs. Hence, we used flotation through a sucrose density gradient to isolate LDs (Ingelmo-Torres et al., 2009) from oleic acid-loaded HepG2 cells that were transiently expressing OPG2-HSD17B11-FLAG.

**Fig. 3.**
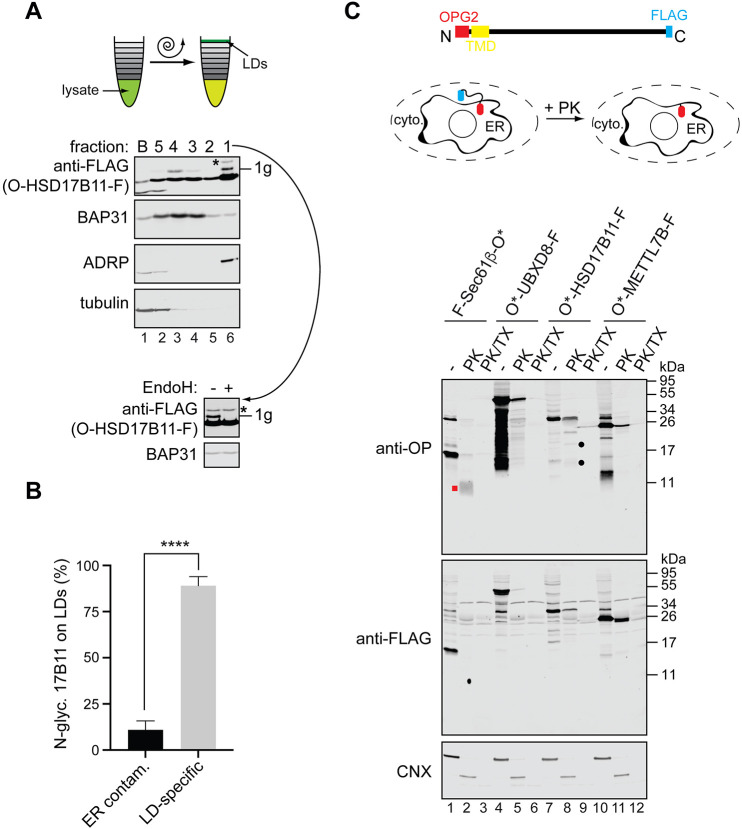
**LD membrane proteins co-translationally inserted into the ER reach LDs.** (A) HSD17B11 with an N-terminal OPG2 tag and a C-terminal FLAG epitope (O-HSD17B11-F) was expressed in HepG2 cells, which were subsequently loaded with oleic acid in the presence of 50 µM zVAD-fmk to inhibit cytosolic N-glycanase (Misaghi et al., 2004). Cells were homogenised, LDs isolated from the post-nuclear supernatant by flotation through a sucrose gradient, and fractions analysed for the presence of selected intracellular compartments by western blotting with the following organelle-specific markers: BAP31 (ER), ADRP (LDs) and tubulin (cytosol). Migration of ectopically expressed OPG2-HSD17B11-FLAG was established by western blotting with the anti-FLAG antibody. ‘1g’ indicates singly N-glycosylated OPG2-HSD17B11-FLAG, and ‘*’ denotes an EndoH-resistant species most likely crossreacting with the anti-FLAG antibody. Inset shows products recovered in fraction 1 with and without EndoH treatment prior to analysis as described. (B) The proportion of N-glycosylated OPG2-HSD17B11-FLAG in the LD fraction (see panel A) that corresponds to contaminating ER membranes and to authentic LD-associated species was calculated based on the relative levels of an ER marker, BAP31, in each fraction (see Materials and Methods). Data are mean±s.e.m. ****P<0.0001 (two-tailed unpaired *t*-test). *n*=4 biological replicates. (C) A schematic representation of the ectopically expressed LD membrane proteins and experimental setup used to address their accessibility to proteinase K (PK). Indicated LD membrane proteins and a control (fully membrane-spanning protein Sec61β) tagged with the FLAG epitope (‘F’) and a variant of the OPG2 tag with mutated N-glycan consensus sites (‘O*’) were transiently expressed in HeLa cells. The plasma membrane was then selectively permeabilised and protease accessibility tested either in the absence or presence of 1% (v/v) Triton X-100 (TX). Samples were resolved by SDS-PAGE and results were visualised by western blotting using anti-rhodopsin (OP) and anti-FLAG antibodies. Western blotting for an endogenous ER membrane protein, calnexin (CNX), was used to compare ER membrane integrity between samples and provide an internal control. Filled red square and black circles indicate candidate membrane-protected protease-resistant fragments.

A clear separation of LDs from the ER was achieved with LDs floating to fraction 1 at the top of the gradient (Fig. 3A, lane 6) and the ER distributed more broadly, with the bulk of it located in fractions 3, 4 and 5 (see Fig. 3A, lanes 2, 3 and 4). The N-glycosylated form of OPG2-HSD17B11-FLAG was located in two distinct fractions, with the strongest signal recovered in the LD-containing fraction 1 (Fig. 3A, lane 6, ‘1g’), together with most of non-glycosylated OPG2-HSD17B11-FLAG. The second pool of N-glycosylated OPG2-HSD17B11-FLAG was found in the middle of the ER-containing fractions (Fig. 3A, lane 3, ‘1g’). In order to establish that the N-glycosylated OPG2-HSD17B11-FLAG recovered in the LD-enriched fraction 1 is authentically LD associated, we calculated the potential ER contamination of this fraction, relative to the peak ER-containing fraction 4, by quantifying their respective levels of BAP31 (also known as BCAP31) (see the Materials and Methods section). Using this criterion, we conclude that the vast majority of N-glycosylated OPG2-HSD17B11-FLAG (∼89%) is authentically bound to LDs, together with a substantial fraction of the non-glycosylated protein. Hence, only ∼11% of the N-glycosylated OPG2-HSD17B11-FLAG recovered in the LD fraction can be ascribed to contamination arising from its ER-localised form (Fig. 3B).

Neither charged amino acids nor hydrophilic sugar moieties are readily accommodated by the hydrophobic interior of LDs (Thiam et al., 2013), and the LD phospholipid monolayer is incompatible with the extended conformation of a ‘classical’ helical transmembrane span (Khaddaj et al., 2022; Thiam et al., 2013). Therefore, we speculate that both the non-glycosylated and N-glycosylated forms of OPG2-HSD17B11-FLAG must assume a hairpin topology in the LD membrane. In the case of the N-glycosylated form of OPG2-HSD17B11-FLAG, for which we know its N terminus has been exposed to the ER lumen, we postulate that its topology must at some stage have been rearranged from an ER bilayer-spanning integral TMD to a LD monolayer-inserted hairpin loop arrangement.

To further test this hypothesis, we carried out a protease protection assay on plasma membrane-permeabilised HeLa cells expressing LD membrane proteins tagged with an OPG2 tag at their N terminus and a FLAG tag at their C terminus (Fig. 3C). As N-glycosylation can influence the transmembrane orientation of proteins at the ER (Goder et al., 1999), we used a modified variant of the OPG2 tag (O*), which is incapable of being N-glycosylated. In parallel, we analysed the protease accessibility of a model ER-resident membrane protein, Sec61β, tagged with the same epitopes, albeit at opposite termini to reflect its membrane topology. We found that proteinase K-digested epitope-tagged Sec61β generates a membrane-protected ER lumenal fragment (Fig. 3C, lanes 1 and 2, red square ‘anti-OP’ panel) that was lost following disruption of the ER membrane with Triton X-100 (Fig. 3C, compare lanes 1-3, ‘anti-OP’ panel). In contrast, although in some cases a fraction of the full-length protein was refractive to any digestion in the absence of detergent, comparable membrane protected fragments were not apparent with the LD membrane proteins tested (Fig. 3C, lanes 4-12, ‘anti-OP’ panel). This behaviour included METTL7B and HSD17B11 that had displayed efficient N-glycosylation at the N terminus in previous assays (Fig. 2A,B, Fig. 3A). This suggests that although the N termini of these proteins are transiently exposed to the ER lumen, where they become accessible to the N-glycosylation machinery, they do not normally reside in a stable membrane-spanning topology at the ER. For O*-HSD17B11-F, some larger proteinase K-protected fragments are faintly visible (Fig. 3C, lanes 7 and 8, filled black circles, ‘anti-OP’ panel), suggesting that a fraction of the newly synthesised protein may not have fully reoriented.

In further support of a model in which a subset of LD membrane proteins can transiently span the ER membrane during their biogenesis, we found that N-glycosylation of METTL7B and HSD17B11 bearing a wild-type OPG2 tag effectively ‘traps’ a fraction of these proteins in a fully membrane-spanning topology. This N-glycan-dependent trapping results in the presence of protease-protected EndoH-sensitive species that are absent without such N-glycosylation (Fig. S5; Fig. 3C, ‘anti-OP’ panels). In contrast, the FLAG epitope placed at the C terminus of the LD membrane proteins was consistently accessible to proteinase K, confirming its cytosolic localisation (Fig. 3C; Fig. S5, ‘anti-FLAG’ panels). On the basis of these data, we conclude that the N termini of newly synthesised LD membrane proteins that follow the co-translational route access the ER lumen only transiently and do not stably reside in a membrane-spanning orientation. However, although the co-fractionation of N-glycosylated OPG2-HSD17B11-FLAG with LDs (Fig. 3A,B) supports this model, we cannot rule out the alternative possibility that two topologically distinct populations of LD membrane proteins are synthesised, and that these two cohorts have distinct fates (see Discussion).

### Membrane-embedded region specifies pathway selection

**Fig. 4.**
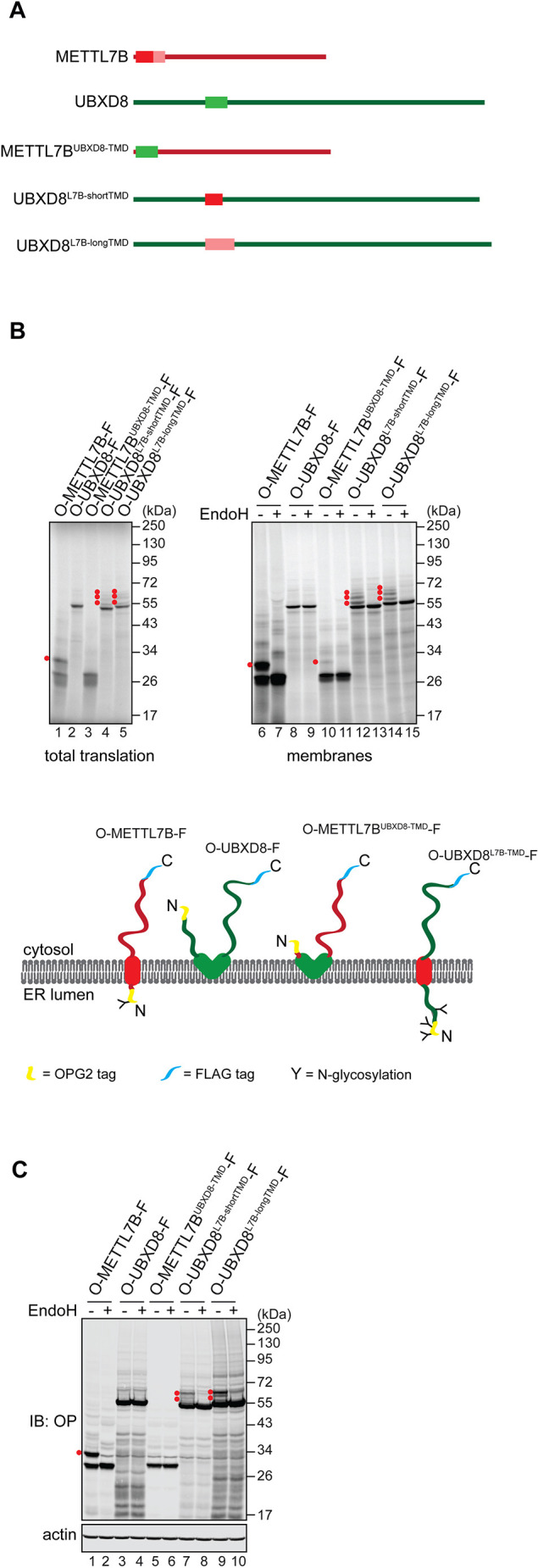
**The membrane-embedded region of LD membrane proteins specifies pathway selection.** (A) A schematic representation of the constructs used in Fig. 4. METTL7B^UBXD8-TMD^: METTL7B with its predicted TMD (residues 3-26, Hessa et al., 2007) replaced with a putative hairpin of UBXD8 (residues 91-119, Zehmer et al., 2009). UBXD8^L7B-shortTMD^: UBXD8 with its hairpin region replaced with residues 3-26 of METTL7B. UBXD8^L7B-longTMD^: UBXD8 with its hairpin region replaced with residues 3-40 of METTL7B. (B) METTL7B and UBXD8 variants (see panel A), tagged with an N-terminal OPG2 (‘O’) and a C-terminal FLAG (‘F’) epitope, were translated *in vitro* in rabbit reticulocyte lysate in the presence of ER-derived microsomes. Membranes were isolated and, where indicated, treated with EndoH, followed by SDS-PAGE and phosphorimaging. Red dots indicate N-glycosylated protein species. Schematic representation of each chimeric protein topology based on our experimental results is also shown (below) using the same colour coding as in A. (C) Protein variants used in B were transiently expressed in U2OS cells, which were then lysed and, where indicated, treated with EndoH. Samples were resolved by SDS-PAGE and results were visualised by western blotting with antibodies against rhodopsin (OP) and actin.

We next investigated what determines LD membrane protein entry into either the co- or post-translational biosynthetic pathway at the ER (Figs 1, 2). We speculated that this choice might depend on the properties of the hydrophobic region, which mediates targeting to and insertion into the ER, and/or the presence of a longer hydrophilic domain on the N-terminal side of this region, as observed for both UBXD8 and HSD17B7 (Fig. 1A). To test this hypothesis, we generated chimaeras between METTL7B and UBXD8, two LD membrane proteins that take distinct routes to the ER (Fig. 1B, Fig. 2A,B). Hence, we replaced the predicted TMD of METTL7B with the hairpin motif of UBXD8 (Fig. 4A, METTL7B^UBXD8-TMD^). In parallel, we substituted UBXD8 hairpin loop with the predicted TMD of METTL7B (UBXD8^L7B-shortTMD^), or with the slightly longer region experimentally shown to mediate both ER membrane insertion and efficient trafficking to LDs [UBXD8^L7B-longTMD^ (Zehmer et al., 2008), see Fig. 4A legend for details of the constructs used]. We then assessed the N-glycosylation of the N-terminal OPG2 tag for each of these constructs.

As previously, OPG2-METTL7B-FLAG was efficiently N-glycosylated, and N-glycosylation of OPG2-UBXD8-FLAG was undetectable both *in vitro* and in cells (Fig. 4B, lanes 6-9, and Fig. 4C, lanes 1-4). Strikingly, N-glycosylation of OPG2-tagged METTL7B was virtually absent when its predicted TMD was replaced with the hairpin motif taken from UBXD8 (Fig. 4B, lanes 10 and 11, and Fig. 4C, lanes 5 and 6). Conversely, substituting the UBXD8 hairpin with either of the two versions of the hydrophobic region from METTL7B resulted in the clear N-glycosylation of these UBXD8 variants (Fig. 4B, lanes 12-15, and Fig. 4C, lanes 7-10). Interestingly, in the cell-free system, we detected a faint band that corresponded to a triply N-glycosylated form of OPG2-UBXD8^L7B-shortTMD^-FLAG and OPG2-UBXD8^L7B-longTMD^-FLAG, despite the OPG2 tag having just two N-glycosylation sites (Fig. 4B, lanes 12-15). This most likely reflects the inefficient N-glycosylation of an endogenous non-consensus Asn residue located in the relatively long N-terminal region of UBXD8, which is translocated to the ER lumen in the chimaeras used (Breitling and Aebi, 2013; Dutta et al., 2017). Taken together, we conclude that biosynthetic pathway selection is primarily determined by the membrane-inserting region of LD membrane proteins.

### Co-translational insertion of LD membrane proteins relies on the EMC

**Fig. 5.**
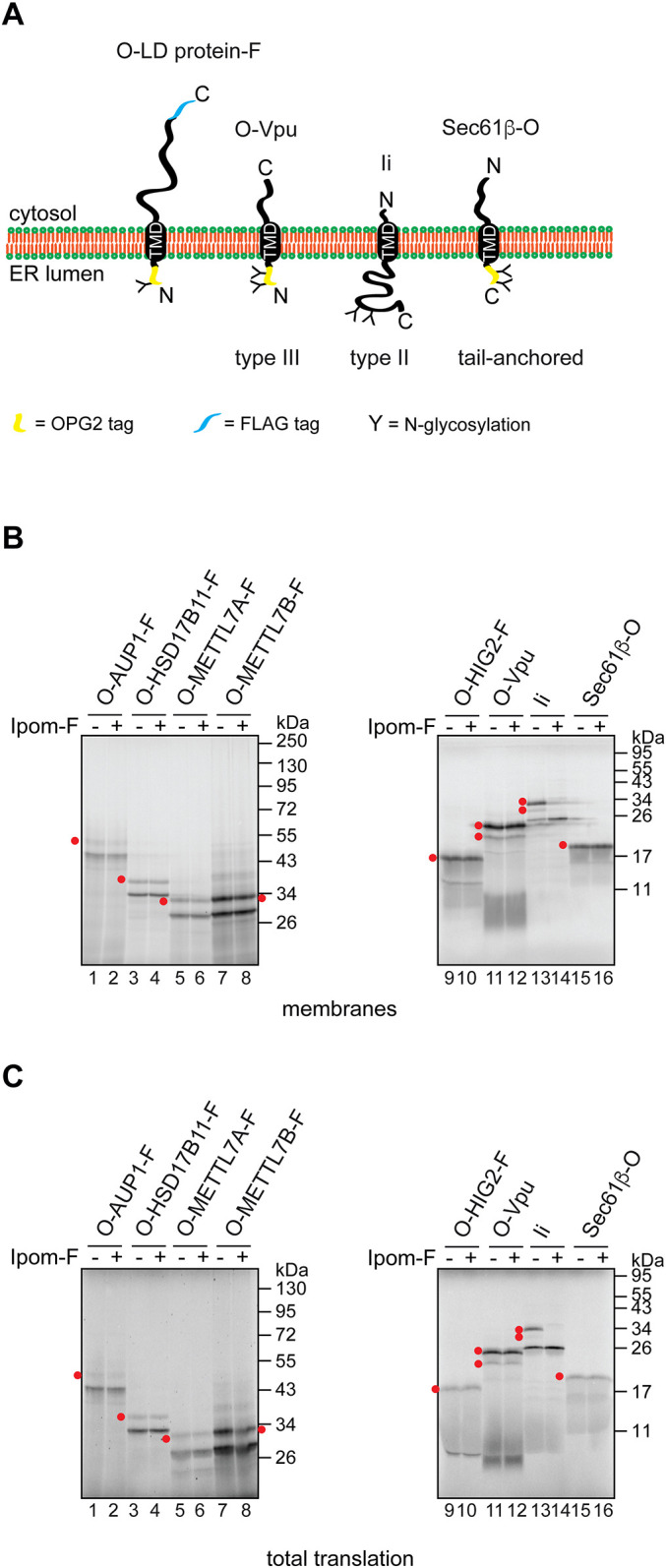
**Insertion of LD membrane proteins into the ER is insensitive to Ipom-F.** (A) Topology of the membrane proteins used in Fig. 5. ‘O’ and ‘F’ indicate the OPG2 and FLAG tags, respectively. (B,C) Indicated membrane proteins were translated *in vitro* in the presence of ER-derived microsomes and 1 µM Ipom-F, a small molecule inhibitor of the Sec61 channel (Zong et al., 2019), or a solvent control. Isolated membranes (B) and total translation reactions (C) were resolved by SDS-PAGE and results were visualised by phosphorimaging. Vpu, Sec61β and Ii were used as control membrane proteins that are insensitive (Vpu and Sec61β) or sensitive (Ii) to Ipom-F (O'Keefe et al., 2021b,c). Red dots indicate N-glycosylated protein species.

To investigate the molecular basis for co-translational insertion of LD membrane proteins into the ER membrane, we took advantage of the small molecule Ipomoeassin F (Ipom-F), which selectively inhibits the Sec61-mediated integration of membrane proteins at the ER (O'Keefe et al., 2021b,c; Zong et al., 2020, 2019). When an *in vitro* membrane insertion assay of OPG2-tagged LD membrane proteins was performed in the presence of Ipom-F, their membrane insertion, as judged by the N-glycosylation of their N-terminal region, was unaffected (see Fig. 5A for topology of the proteins used, and Fig. 5B,C, lanes 1-10). In contrast, the N-glycosylation of a classical Sec61 substrate, the type II membrane protein Ii, was almost completely blocked (Fig. 5B,C, lanes 13 and 14), in line with previous studies (Zong et al., 2020, 2019). Hence, the behaviour of these LD proteins mirrors that of clients that do not require the insertase activity of the Sec61 complex for their integration (Fig. 5B,C, lanes 11 and 12, and 15 and 16), namely Vpu, a type III membrane protein which uses the EMC (O'Keefe et al., 2021c), and Sec61β, a tail-anchored protein that can access multiple pathways for membrane insertion at the ER (Casson et al., 2017; Guna et al., 2018). On this basis, we concluded that the translocation of the N termini of LD membrane proteins into the ER lumen does not require translocation via the Sec61 complex.

When LD proteins insert into the ER membrane with their N-terminal extension located inside the ER lumen they assume, albeit transiently, the same orientation as a stable type III transmembrane protein, such as the Vpu protein (Fig. 5A; see also McKenna et al., 2017; O'Keefe et al., 2021b,c). As bona fide type III membrane proteins are integrated via a novel pathway that utilises the membrane insertase activity of the EMC (Chitwood et al., 2018; O'Keefe et al., 2021c), we explored the possibility that some LD membrane proteins may also utilise the EMC during their membrane insertion. To this end, we carried out *in vitro* translation reactions in the presence of semi-permeabilised cells depleted of specific ER components implicated in membrane protein biogenesis (Lang et al., 2012; O'Keefe et al., 2021b,c). Briefly, HeLa cells transfected with siRNA oligonucleotides targeting specific membrane components were treated with digitonin to selectively permeabilise their plasma membrane, the cytosol was washed away and the resulting semi-permeabilised cells used as a source of membranes for *in vitro* translation reactions (Wilson et al., 1995). On the basis of our present findings and those of previous studies (O'Keefe et al., 2021b,c; Schrul and Kopito, 2016) we chose to deplete the following: SRα, a subunit of the SRP receptor that enables the co-translational delivery of nascent precursor proteins to the ER membrane (O'Keefe et al., 2021a); EMC5 (also known as MMGT1), a core structural subunit of the EMC (Bai et al., 2020; Miller-Vedam et al., 2020; O'Donnell et al., 2020; Pleiner et al., 2020; Volkmar et al., 2019); Sec61α, a central component of the Sec61 translocon (O'Keefe et al., 2021a); and Pex3, a membrane-tethered component of the post-translational pathway used by UBXD8 (Schrul and Kopito, 2016).

**Fig. 6.**
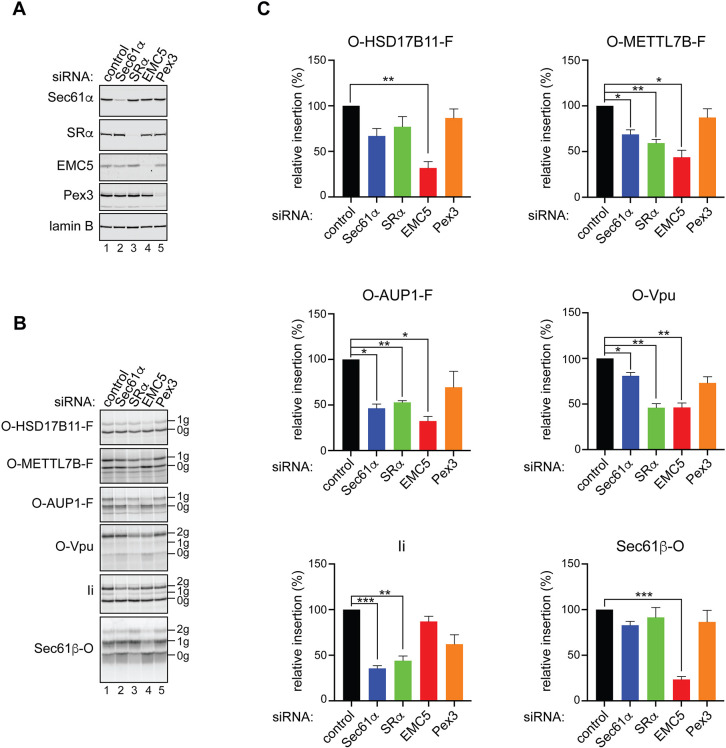
**The EMC facilitates the biogenesis of LD membrane proteins at the ER.** (A) HeLa cells were depleted of selected factors implicated in membrane protein biogenesis at the ER via siRNA-mediated knockdowns, their plasma membranes selectively permeabilised and knockdown efficiency confirmed by western blotting with antibodies against the indicated proteins (see also Table S1). (B) The semi-permeabilised HeLa cells (see panel A) were used as a source of ER membrane during *in vitro* synthesis of the indicated LD membrane proteins tagged with the OPG2 epitope (‘O’) at the N terminus and the FLAG tag (‘F’) at the C terminus. In parallel, OPG2-Vpu, an EMC-dependent type III membrane protein (O'Keefe et al., 2021b,c), the tail-anchored protein Sec61β-OPG2 and Ii, a classical Sec61-dependent substrate, were synthesised. Membrane fractions were isolated, resolved by SDS-PAGE and results visualised by phosphorimaging. ‘0g’ indicates non-glycosylated, ‘1g’ singly N-glycosylated and ‘2g’ doubly N-glycosylated protein species. (C) ER membrane insertion efficiency, defined as the amount of N-glycosylated species in the membrane fraction and normalised to values obtained for the non-targeting siRNA, was calculated for each of the proteins and knockdown conditions. Data are mean±s.e.m. *P≤0.05, **P≤0.01, ***P≤0.001 (repeated measures one-way ANOVA with pairwise comparisons carried out using Dunnett's multiple comparisons test). *n*≥3 biological replicates.

Having confirmed ∼85-96% depletion of each of these membrane-localised factors (Fig. 6A; Table S1), we then tested how their loss affects the ER insertion of OPG2-tagged LD membrane proteins by comparing the amount of N-glycosylated products that were recovered relative to control semi-permeabilised cells (Fig. S6). Perturbation of the EMC dramatically reduced the amount of N-glycosylated, and hence fully membrane-spanning, polypeptides for all three co-translationally inserting LD membrane proteins tested (Fig. 6B, compare 1 g species in lanes 1 and 4 for OPG2-HSD17B11-FLAG, OPG2-METTL7B-FLAG and OPG2-AUP1-FLAG). In contrast, the insertion of Ii, a Sec61-dependent type II membrane protein (Fig. 5A), was barely altered (Fig. 6B, 2 g species in lanes 1 and 4 for Ii). Quantification showed that EMC5 depletion reduced the integration of the three model LD membrane proteins that employ the co-translational pathway by >50% (Fig. 6C), an outcome that is directly comparable to the defect in membrane insertion observed with OPG2-Vpu, a bone fide type III membrane protein recently shown to utilise the EMC (O'Keefe et al., 2021c; Fig. 5A). Likewise, depletion of the SRP receptor, which acts upstream of the EMC insertase during the co-translational insertion of type III proteins (O'Keefe et al., 2021c), also significantly reduced the membrane insertion of two LD proteins, OPG2-METTL7B-FLAG and OPG2-AUP1-FLAG, the type III membrane protein OPG2-Vpu and the classical co-translational Sec61 client Ii (Fig. 6C). In contrast, although the membrane insertion of Sec61β-OPG2, a post-translational tail-anchored protein client of the EMC (Guna et al., 2018), showed the strongest reduction of any protein tested following EMC5 depletion, loss of SRα had no significant effect *in vitro* (Fig. 6C). Hence, in contrast to such tail-anchored proteins, LD protein clients that employ the EMC co-translationally also require the actions of the SRP receptor, as previously reported for bona fide type III membrane proteins (O'Keefe et al., 2021c).

Interestingly, although the membrane insertion of LD membrane proteins via this co-translational pathway is insensitive to the Sec61 inhibitor Ipom-F (Fig. 5), two of these proteins, OPG2-METTL7B-FLAG and OPG2-AUP1-FLAG, showed a significant loss of membrane insertion following Sec61α depletion (Fig. 6C). This mirrors the recently reported behaviour of type III membrane proteins (O'Keefe et al., 2021c; see also Fig. 6C, OPG2-Vpu), and suggests that the Sec61 translocon may contribute to LD membrane protein biogenesis via a mechanism that does not require its insertase activity (see Discussion). Despite the effectiveness of its knockdown (∼90%, see Table S1), Pex3 depletion had no effect on the membrane insertion of any of the proteins tested (Fig. 6C), consistent with its role as a receptor for the post-translational insertion of LD proteins with preformed hairpin loops into the cytosolic leaflet of the ER membrane (Schrul and Kopito, 2016). In summary, our studies suggest that a subset of LD membrane proteins is synthesised via an alternative co-translational pathway that initially involves their EMC-mediated insertion as ‘type III-like’ membrane proteins. Unlike bona fide type III membrane proteins, these LD membrane proteins are subsequently able to dislocate their short hydrophilic N termini into the cytosol, thereby generating a stable hairpin loop conformation that is competent for incorporation into newly forming LDs.

## DISCUSSION

LD membrane proteins regulate key aspects of LD function and dynamics (Olzmann et al., 2013; Wilfling et al., 2013; Zhang et al., 2017), and have been directly linked to cancer (VandeKopple et al., 2019; Zhang et al., 2017) and viral propagation (Park et al., 2015; Zhang et al., 2018). They initially insert into the ER before being trafficked to LDs (Ingelmo-Torres et al., 2009; Ostermeyer et al., 2004; Schrul and Kopito, 2016; Stevanovic and Thiele, 2013; Turro et al., 2006; Zehmer et al., 2009, 2008). Early studies using heterologous systems suggested that plant oleosins, authentic LD membrane proteins, and mammalian caveolins, which localise to LDs under specific conditions, associate with the ER membrane co-translationally in a process mediated by SRP, SR and, for caveolins, the Sec61 translocon (Abell et al., 2002; Beaudoin et al., 2000; Monier et al., 1995). However, a more recent report (Schrul and Kopito, 2016) established that the model LD membrane protein UBXD8 is delivered to the ER post-translationally via a pathway that is mediated by Pex19 and its ER membrane-localised receptor, Pex3. This Pex19/Pex3-dependent pathway is also suggested to accommodate other hairpin-forming ER-resident proteins (Yamamoto and Sakisaka, 2018), and hence was proposed as a general pathway for the ER membrane insertion of LD-destined membrane proteins (Dhiman et al., 2020; Schrul and Kopito, 2016; Yamamoto and Sakisaka, 2018).

**Fig. 7.**
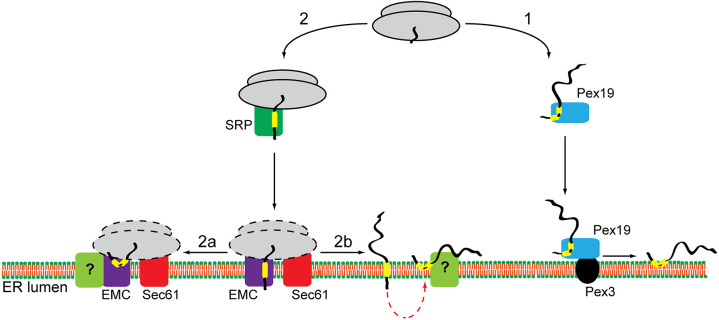
**Model for LD membrane protein biogenesis at the ER.** Depending on the properties of their hydrophobic membrane-inserting region, LD membrane proteins enter one of two potential biosynthetic pathways. Proteins, such as UBXD8 and HSD17B7, are released into the cytosol, bound by Pex19 and post-translationally delivered to the ER (route 1). Interaction between Pex19 and its membrane-receptor Pex3 releases such LD membrane proteins in a hairpin conformation into the ER membrane. Alternatively, LD membrane proteins, such as HSD17B11, METTL7A, METTL7B, AUP1 and HIG2, are delivered co-translationally to the ER and insert into the lipid bilayer in a reaction facilitated by the EMC with additional insertase-independent contributions from the Sec61 complex (route 2). At present, it is unclear exactly how the EMC and Sec61 complex cooperate, and which complex facilitates ribosomes binding (indicated by dashed lines). We speculate that the co-translationally delivered LD membrane proteins transiently expose their N terminus to the ER lumen but then re-arrange their topology to form a hairpin with both termini facing the cytosol, which is a prerequisite for trafficking to LDs. Such topological reorientation could occur either co-translationally (route 2a) or following complete protein synthesis (route 2b). At present, it is unknown whether this process is spontaneous or requires an assistance from a dedicated factor(s) (indicated with ‘?’).

Here, we have used a homologous mammalian cell-free translation system together with cell-based studies to investigate the biogenesis of a panel of well-defined human LD membrane proteins at the ER. We find that LD membrane proteins can be divided into at least two different groups, which show distinct requirements for delivery to and insertion into the ER (Fig. 7). The first group is exemplified by UBXD8 and HSD17B7, and follows a post-translational targeting pathway as defined previously (Schrul and Kopito, 2016; Fig. 1B). The second group relies on a co-translational route for their efficient delivery to the ER, and its clients include AUP1, HSD17B11, METTL7A, METTL7B and HIG2 (Fig. 1B). The biogenesis of these co-translationally inserted LD membrane proteins is mediated by the EMC, with additional contributions from SR and the Sec61 translocon detected for METTL7B and AUP1 (Fig. 6). The ER insertion of such LD membrane proteins is consistently insensitive to Ipom-F (Fig. 5), a small molecule inhibitor of the Sec61 translocon (Zong et al., 2019), which suggests that it is not acting as an insertase in this context. Rather, we speculate that the Sec61 complex can enhance the co-translational insertase activity of the EMC, as recently established for the biogenesis of bona fide type III membrane proteins (O'Keefe et al., 2021c).

Our data suggest that the EMC may not discriminate between proteins that stably reside in a membrane-spanning topology and proteins that can subsequently acquire a hairpin conformation, consistent with the ability of the EMC to translocate both N-terminal and C-terminal segments of hydrophilic polypeptide that are adjacent to a hydrophobic transmembrane region (Bai et al., 2020; Chitwood et al., 2018; Guna et al., 2018; Miller-Vedam et al., 2020; O'Donnell et al., 2020; Pleiner et al., 2020). Our finding that co-translationally inserted LD membrane proteins can typically expose their short N termini to the ER lumen (Fig. 2) confirms previous studies using chimeric LD proteins with artificial N-terminal signal sequences. Hence, signal sequence bearing versions of METTL7A (Zehmer et al., 2009) and AUP1 (Stevanovic and Thiele, 2013) are efficiently targeted to the ER and processed by ER lumenal signal peptidase (Paetzel et al., 1998), and the resulting signal sequence-cleaved proteins are incorporated into LDs (Stevanovic and Thiele, 2013; Zehmer et al., 2009). Likewise, in this study, we show that N-glycosylated HSD17B11 can reach mature LDs (Fig. 3A,B). Hence, exposure of the N termini of such LD proteins to the ER lumen, whether by virtue of an artificial signal sequence (Stevanovic and Thiele, 2013; Zehmer et al., 2009) or not (this study), does not prohibit either their acquisition of a hairpin topology or their authentic LD localisation. Taken together, these studies support the idea that the co-translational biogenesis of LD membrane proteins at the ER generates a functional pool of polypeptides that can be trafficked to LDs rather than an ‘off-target’ pathway. It should be noted that on the basis of our current data we cannot formally exclude the possibility that two distinct populations of LD proteins are synthesised via this co-translational route: one population that immediately assumes a hairpin conformation on the cytosolic leaflet of the ER membrane and subsequently enters LDs, and a second that spans the membrane with its N terminus in the ER lumen but is unable to acquire a hairpin topology. In the latter case, such proteins may be recognised as aberrant or mis-inserted, resulting in their ER-associated degradation (Wu and Rapoport, 2018).

At present, our favoured model for the EMC-mediated co-translational biogenesis of LD proteins incorporates the possibility that newly synthesised polypeptides may reorient from a fully membrane-spanning topology to a hairpin one, in order to be accommodated by LDs (Abell et al., 2002; Stevanovic and Thiele, 2013; Zehmer et al., 2009, 2008). The normally transient nature of such N-terminal domain residency in the ER lumen is supported by our protease protection studies. Hence, we preferentially detected discrete membrane-protected N-terminal fragments of LD membrane proteins in the presence of N-linked glycans (Fig. 3C; Fig. S5), which most likely ‘trap’ such otherwise labile topological intermediates (Goder et al., 1999). At present, we can only speculate as to how the reorientation of such LD membrane proteins might occur. One possibility is that the TMD acquires its hairpin conformation co-translationally (Fig. 7, route 2a). This would result in the brief exposure of the N terminus to the ER lumen, consistent with the incomplete N-glycosylation of most LD-membrane proteins that we observed (Fig. 2). In this scenario, LD membrane protein biogenesis would resemble that of some type II membrane proteins that initially insert ‘head-first’ (N terminus translocated) and subsequently completely invert their topology within the Sec61 translocon so that their N terminus faces the cytosol (Devaraneni et al., 2011; Goder and Spiess, 2003). Alternatively, LD membrane proteins might complete their synthesis, be released into the ER membrane and only then reorient their topology (Fig. 7, route 2b), as reported for some bacterial membrane proteins in response to changes in the membrane lipid composition (Dowhan et al., 2019).

How LD membrane proteins might transition from a fully membrane-spanning topology to a hairpin one, and whether this process is spontaneous or requires dedicated factors, are key questions for future studies. Interestingly, when the hairpin motif of a LD membrane protein is replaced with a ‘classical’ TMD, the resulting polypeptide does not localise to LDs (Zehmer et al., 2008), and the ER-LD interface appears to act as a barrier that can exclude fully membrane-spanning proteins from LDs (Khaddaj et al., 2022). In this study, we show that the TMD of METTL7B can support the translocation of ∼90 N-terminal residues of UBXD8 into the ER lumen, and when UBXD8 hairpin is incorporated into METTL7B it almost completely abolishes the otherwise efficient translocation of its much shorter N terminus (Fig. 4). Therefore, it seems likely that, in addition to features such as hydrophobicity, the folding of the TMD/hairpin loop region may also influence which biosynthetic route is taken (Fig. 7); for example, by impacting on the recruitment of targeting factors or the ability of a particular client to engage the EMC. Although our study indicates that the TMD/hairpin motif is the primary determinant for pathway selection, it is possible that specific sequence features of its flanking regions can influence the efficiency of pathway entry. For example, replacing the ∼100 residues located N-terminally to the TMD/hairpin of caveolin-1 with a region of a comparable length derived from rat growth hormone changes the topology of a fraction of the protein from a hairpin loop to fully membrane spanning (Monier et al., 1995). It should also be noted that our studies of RDH14 indicate that at least some LD proteins can use both routes effectively, suggesting that these pathways are not mutually exclusive but rather that some clients are more dependent on one or other, as also reported for the biogenesis of tail-anchored proteins at the ER (Casson et al., 2017).

In summary, we show that LD membrane proteins can be delivered to the ER via at least two distinct pathways that can most simply be classified as co-translational (this study) and post-translational (Schrul and Kopito, 2016). Our work reconciles some apparent discrepancies between previous publications (Abell et al., 2002; Beaudoin et al., 2000; Monier et al., 1995; Schrul and Kopito, 2016) and lays the foundations for future studies aimed at unravelling the complexity of LD membrane protein biogenesis that we have revealed.

## MATERIALS AND METHODS

### Materials

LD membrane protein cDNAs in pcDNA3.1+/C-(k)DYK were purchased from GenScript and their variants with the OPG2 tag, AUP1 point mutants and METTL7B/UBXD8 chimaeras were generated by site-directed mutagenesis. Sec61β with a C-terminal OPG2 tag in pcDNA5, either with or without an N-terminal FLAG epitope, has been described previously (Leznicki and High, 2020). Nuclease-treated rabbit reticulocyte lysate was obtained from Promega (L4960), [^35^S] methionine (EasyTag EXPRESS ^35^S Protein Labelling Mix) from PerkinElmer, LipidToxRed stain (H34476) from Thermo Fisher Scientific and EndoH (P0702 and P0703) from New England Biolabs. The hybridoma line producing the monoclonal anti-rhodopsin antibody (1:1000) (Adamus et al., 1991) was provided by Paul Hargrave (Department of Ophthalmology, University of Florida, USA), monoclonal anti-tubulin antibody (1:1000) by Keith Gull (University of Oxford, UK), rabbit polyclonal anti-Sec61α antibody (1:1000) was a gift from Richard Zimmermann and Sven Lang (Saarland University, Homburg, Germany), and rabbit anti-SRα antibodies (1:1000) (Jadhav et al., 2015) were a gift from Martin Pool (University of Manchester, UK). The following commercial antibodies were used: rabbit anti-β-actin (Abcam, ab8227, 1:5000, batch numbers GR3176830-1 and GR3224338-1); mouse anti-FLAG (clone M2, Sigma-Aldrich, F3165, 1:2500, batch number SLBH1191V); rabbit anti-FLAG (Sigma-Aldrich, F7425, 1:1000); anti-AUP1 (Bethyl Laboratories, A302-899A, 1:1000, batch number 1); anti-BAP31 (ProteinTech, 11200-1-AP, 1:1000, batch number 00018537); anti-ADRP (Abcam, ab78920, 1:1000, batch number GR3227109-1); anti-calnexin (Cell Signaling Technology, clone C5C9, 2679S, 1:2000, batch number 4); anti-MMGT1 (EMC5) (Bethyl Laboratories, A305-832A-M, 1:1000, batch number 1); anti-Pex3 (St John's Laboratory, STJ29491, 1:1000, batch number 949135460201); and anti-lamin B (SantaCruz Biotechnology, sc-6217, 1:1000, batch number J1311). MMGT1 (EMC5) siRNA was obtained from Thermo Fisher Scientific (s41129), whereas all the other siRNAs were made to order as ‘on-target+’ by Horizon Discovery. U2OS cells were procured from the European Collection of Authenticated Cell Cultures, whereas HeLa and HepG2 cells were obtained from the American Type Culture Collection, and all were checked for mycoplasma infection. Ipom-F was synthesised as described previously (Zong et al., 2015, 2020, 2017).

### *In vitro* transcription and translation

Templates for *in vitro* transcription were generated by PCR, mRNA was prepared as described previously (Leznicki et al., 2013) and used in *in vitro* translation reactions comprising rabbit reticulocyte lysate, 1 mCi/ml [^35^S] methionine and amino acid mix lacking methionine. Where indicated, reactions were also supplemented with 1 µM Ipom-F or an equal volume of DMSO solvent. To compare co- and post-translational insertion into the ER, LD membrane proteins and control proteins were synthesised in parallel, either in the presence or absence of ER-derived dog pancreatic microsomes at 30°C for 7 min, and further translation initiation was blocked with 0.1 mM aurintricarboxylic acid (Alfa Aesar, A15905). Samples were incubated at 30°C for a further 8 min and puromycin (Sigma-Aldrich, 540222) was added to a final concentration of 2.5 mM, and reactions were kept at 30°C for 7 min. At this stage the ‘post-translational’ reactions were supplemented with ER-derived microsomes, and the ‘co-translational’ reactions were supplemented with an equal volume of KHM buffer [110 mM KOAc, 2 mM Mg(OAc)_2_ and 20 mM HEPES-KOH (pH 7.5)], and all samples were kept at 30°C for 15 min. Aliquots (∼7%) were taken to check total translation products, whereas the remaining samples were spun through a high-salt sucrose cushion [0.75 M sucrose, 0.5 M KOAc, 5 mM Mg(OAc)_2_ and 50 mM HEPES-KOH (pH 7.9)] at 100,000 ***g*** for 10 min at 4°C to isolate the membranes and membrane-associated material. The pellets were then directly resuspended in SDS sample buffer.

To check N-glycosylation of *in vitro* synthesised proteins, translations were carried out at 30°C for 15 min. Further translation initiation was blocked with 0.1 mM aurintricarboxylic acid and samples were incubated at 30°C for 30 min. Total translation aliquots were taken, membranes were isolated as described above and lysed directly in SDS samples buffer, and proteins were denatured at 37°C for 30 min. Samples were then split in two and either buffer control or ∼20,000 U/ml of EndoH variant added, followed by incubation at 37°C for at least 2 h.

All samples were resolved by SDS-PAGE and results visualised by phosphorimaging using a Typhoon FLA 7000 phosphorimager (GE Healthcare). Images were processed and band intensity quantified using AIDA software (Raytek).

### Cell culture and preparation of semi-permeabilised cells

All cells were cultured in Dulbecco's modified Eagle's medium (DMEM, Sigma-Aldrich, D5796) supplemented with 10% (v/v) fetal bovine serum and were maintained in a 5% CO_2_ humidified incubator at 37°C. Transient transfection of U2OS and HepG2 cells with plasmid DNA was carried out using GeneJuice (Merck Millipore, 70967) according to the manufacturer's instructions and keeping GeneJuice to a DNA ratio of 3:1 (volume in µl: weight in µg). To estimate the N-glycosylation of the proteins studied, U2OS cells grown in six-well plates were transfected with 2 µg of plasmid DNA and lysed directly in SDS sample buffer 24 h post transfection. The amount of DNA used for other experiments is described in detail in the relevant sections of Materials and Methods (see below). Depletion of membrane components was carried out in HeLa cells using INTERFERin (Polyplus, 409-10) as a transfection reagent according to the manufacturer's instructions. The following siRNA oligonucleotides were used at 20 nM final concentration: non-targeting siRNA (5′-UGGUUUACAUGUUGUGUGAuu-3′); SEC61A1 (Sec61α) siRNA (5′-AACACUGAAAUGUCUACGUUUuu-3′); SRPRA (SRα) siRNA (5′-GAGCUUGAGUCGUGAAGACuu-3′); PEX3 siRNA (5′-GGGAGGAUCUGAAGAUAAUAAGUUUuu-3′); and MMGT1 (EMC5) siRNA (Thermo Fisher Scientific, s41129). A total of 850,000 cells were plated in a 10-cm dish, transfected with siRNA oligonucleotides the next day and grown for another 72 h, at which point semi-permeabilised cells were prepared. To this end, cells were harvested by trypsinisation in 3 ml 0.25% trypsin-EDTA solution (Sigma-Aldrich, T3924), which was then inhibited by adding 4 ml of ice-cold KHM buffer supplemented with 100 μg/ml soybean trypsin inhibitor (Sigma-Aldrich, T6522). Cells were pelleted at 500 ***g*** for 3 min at 4°C, resuspended in 4 ml ice-cold KHM buffer supplemented with 80 μg/ml high purity digitonin (Calbiochem, 300410) and incubated on ice for 5 min to permeabilise the plasma membrane. Cells were diluted to 14 ml with ice-cold KHM buffer, pelleted at 500 ***g*** for 3 min at 4°C, resuspended in 5 ml ice-cold HEPES buffer [90 mM HEPES-KOH (pH 7.5) and 50 mM KOAc] and incubated on ice for 10 min. Cells were pelleted by centrifugation once more, resuspended in 100 ul KHM buffer and endogenous mRNA was removed by treatment with 0.2 U Nuclease S7 Micrococcal nuclease from *Staphylococcus aureus* (Sigma-Aldrich, 10107921001) in the presence of 1 mM CaCl_2_ at 22°C for 12 min. Nuclease was inactivated by the addition of EGTA to a final concentration of 4 mM, and semi-permeabilised cells were centrifuged at 13,000 ***g*** for 1 min and resuspended in KHM buffer to a final concentration of 3×10^7^ cells/ml. *In vitro* translation reactions were carried out for the indicated proteins using rabbit reticulocyte lysate and semi-permeabilised cells at a final concentration of 3×10^6^ cells/ml at 30°C for 40 min in a thermomixer (Eppendorf) set to 900 rpm. Reactions were placed on ice and 10% (v/v) kept as an input material, with the rest diluted with 1 ml ice-cold KHM buffer, and the semi-permeabilised cells isolated by centrifugation (21,000 ***g***, 2 min, 4°C), followed by lysis in SDS sample buffer.

### Isolation of LDs

LDs were isolated based on the protocol by Ingelmo-Torres et al. (2009), with minor modifications. HepG2 cells in a 15-cm dish were transfected at ∼50% confluency with 10 µg of pcDNA3.1+/C-(k)DYK plasmid encoding OPG2-HSD17B11-FLAG. LD formation was induced 24 h post transfection by adding fresh medium supplemented with 0.5 mM oleic acid (Sigma-Aldrich, O1383) complexed with bovine serum albumin (BSA, Sigma-Aldrich, A8806) (Brasaemle and Wolins, 2016), and zVAD-fmk (Selleck Chemicals, S7023) was added to a final concentration of 50 µM to inhibit N-glycanase (Misaghi et al., 2004). After 16 h, cells were washed twice with ice-cold PBS, harvested by scraping, pelleted (500 ***g***, 5 min, 4°C) and resuspended in 0.5 ml buffer L [50 mM Tris-Cl (pH 7.5), 150 mM NaCl and 5 mM EDTA] supplemented with a complete protease inhibitor cocktail (Sigma-Aldrich, P8340). Cells were broken by passing them 30 times through a cell homogeniser (Isobiotec, Germany) with a tungsten carbide ball of 14 µm clearance. Cell lysate was pre-cleared (1600 ***g***, 5 min, 4°C), and 0.5 ml of supernatant was mixed with 0.5 ml of 2.5 M sucrose in buffer L, and overlaid with 200 µl of 30%, 25%, 20%, 15%, 10% and 5% (w/v) sucrose in buffer L. Samples were centrifuged at ∼166,000 ***g*** for 3 h at 4°C using a TLS-55 rotor (Beckman Coulter) in an Optima benchtop ultracentrifuge (Beckman Coulter) with acceleration set to 9 and deceleration set to 0. Five fractions (280 µl each) were collected from the top using a Hamilton syringe followed by the final ∼700 µl bottom fraction, and an aliquot of each fraction was mixed directly with SDS sample buffer and resolved by SDS-PAGE for western blotting analysis.

To quantify N-glycosylated OPG2-HSD17B11-FLAG in the LD fraction that is authentically LD localised (*Ngly*.17*B*11_*LD specific*_) or ER associated (*Ngly*.17*B*11_*ER contaminants*_) the following formulas were used:

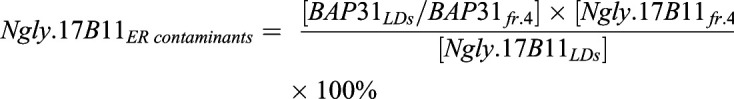
and

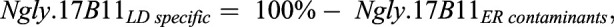
where [*Ngly*.17*B*11_*LDs*_] corresponds to total N-glycosylated OPG2-HSD17B11-FLAG in the LD fraction, [*Ngly*.17*B*11_*fr*.4_] corresponds to total N-glycosylated OPG2-HSD17B11-FLAG in fraction 4 (main ER fraction) and [*BAP*31_*LDs*_/*BAP*31_*fr*.4_] is the ratio between BAP31 levels in the LD fraction and fraction 4.

### Protease protection assay

HeLa cells grown in 10-cm dishes were transfected with 5 µg of the indicated LD membrane protein variants in pcDNA3.1+/C-(k)DYK or Sec61β in pcDNA5, and 24 h post transfection, semi-permeabilised cells were prepared as described above (‘Cell culture and preparation of semi-permeabilised cells’ section) but omitting the nuclease treatment step. Semi-permeabilised cells were resuspended to a final concentration of ∼2.5×10^7^ cells/ml and split into three aliquots, which received the following: water, 1 mg/ml proteinase K (Sigma-Aldrich, P2308) only or 1 mg/ml proteinase K together with 1% (w/v) Triton X-100. Reactions were incubated for 1 h at 22°C in a thermomixer (Eppendorf), with mixing set to 1000 rpm, protease was inhibited with 2.5 mM phenylmethylsulfonyl fluoride and samples were incubated for another 10 min (22°C, 1000 rpm). Reactions were stopped by adding hot SDS sample buffer and immediate incubation at 95°C for 10 min. To reduce the viscosity of samples, DNA was sheared using a BioRuptor (Diagenode).

### Fluorescence microscopy

U2OS cells were grown on glass coverslips in six-well plates, and at ∼40% confluency transfected with 1 µg of plasmids encoding the indicated proteins using GeneJuice as described above. Medium was replaced ∼6 h post-transfection with fresh DMEM supplemented with 0.25 mM oleic acid complexed with BSA (Brasaemle and Wolins, 2016), and the cells were grown for another 16 h. Cells were fixed with 4% paraformaldehyde (w/v) for 15 min at room temperature, washed three times with PBS supplemented with 100 mM glycine (pH 8.0) (5 min each wash step) and finally washed with PBS without glycine. At this stage, their plasma membrane was permeabilised with 20 µM digitonin in PBS for 5 min at room temperature, coverslips were washed three times with PBS and then blocked with 1% BSA (w/v) in PBS for 15 min at room temperature and washed once again with PBS. Coverslips were then incubated with the anti-FLAG antibody [clone M2, 1:800 dilution in 1% (w/v) BSA in PBS] for 1 h at room temperature, washed three times with PBS (5 min each wash step) and incubated for 1 h at room temperature with a secondary donkey anti-mouse IgG antibody conjugated to Alexa Fluor 488 dye [1:1000 dilution in 1% (w/v) BSA in PBS]. After washing three times with PBS (5 min each wash step), the coverslips were incubated with LipidToxRed dye (1:400 in PBS) for 1 h at room temperature, and briefly washed and mounted using ProLong Gold (Thermo Fisher Scientific, P36930). Images were acquired using an Olympus IX83 inverted microscope using a 60×/1.42 Plan Apo objective and a charged-coupled device camera with a Z optical spacing of 0.2 µm. Raw image deconvolution was carried out using Huygens Pro software (SVI) and images were processed using ImageJ (Fiji).

### Statistical analysis

Radiolabelled protein species were quantified using AIDA software, whereas signals resulting from western blotting were quantified using ImageStudioLite (LiCor). Calculations were carried out in Microsoft Excel, and GraphPad Prism was then used to generate graphs and quantify statistical significance using the indicated tests.

## Supplementary Material

Supplementary information

Reviewer comments
