# Supplementary material for: Co-translational biogenesis of lipid droplet integral membrane proteins
Source: J Cell Sci. 2021 Nov 2;135(5):jcs259220. doi: 10.1242/jcs.259220 (PMC8627552; doi:10.1242/jcs.259220)
Supplement: Supplementary information [file joces-135-259220-s1.pdf]

Figure S1

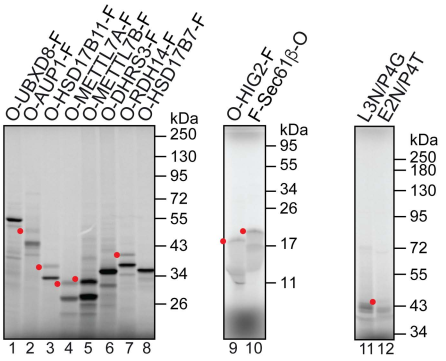

Fig. S1. LD membrane proteins expose their N-termini to the ER lumen.

Indicated LD membrane proteins bearing an N-terminal OPG2 epitope (“O”) and a C-terminal FLAG tag (“F”), FLAG-Sec61β-OPG2 and point mutants of AUP1 (L3N/P4G and E2N/P4T) lacking the OPG2 tag were translated *in vitro* in the presence of ER-derived microsomes. Shown are aliquots of the total translation reactions, which were resolved by SDS-PAGE and visualised by phosphorimaging. The corresponding membrane fraction is shown in Figs 2A and 2C of the main text. Red dots indicate N-glycosylated protein species.

Figure S2

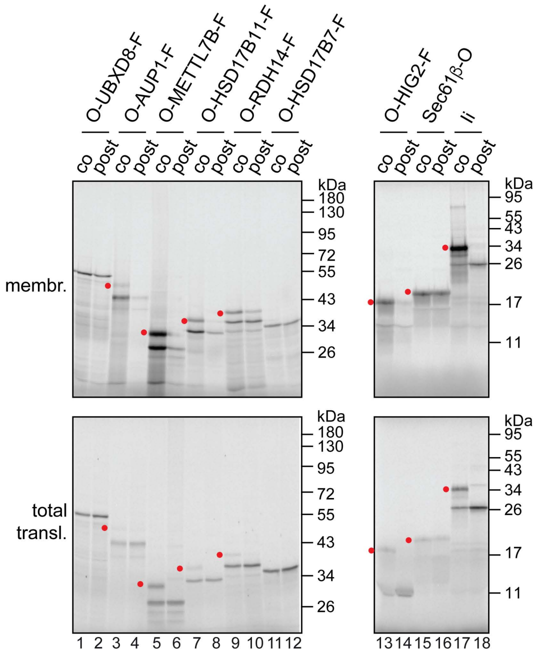

Fig. S2. N- and C-terminal epitope tags do not affect biosynthetic pathway selection by LD membrane proteins.

Co- and post-translational requirements for ER membrane insertion of the indicated LD membrane proteins with an N-terminal OPG2 tag ("O") and a C-terminal FLAG epitope ("F") was tested as for Fig. 1B of the main text. Sec61β with a C-terminal OPG2 tag and untagged invariant chain (Ii) were used as control proteins that rely on the post- and co-translational pathways for delivery to the ER, respectively. Total translation reactions (bottom panels) and the membrane fractions (top panels) are shown. Red dots indicate N-glycosylated protein species.

Journal of Cell Science • Supplementary information

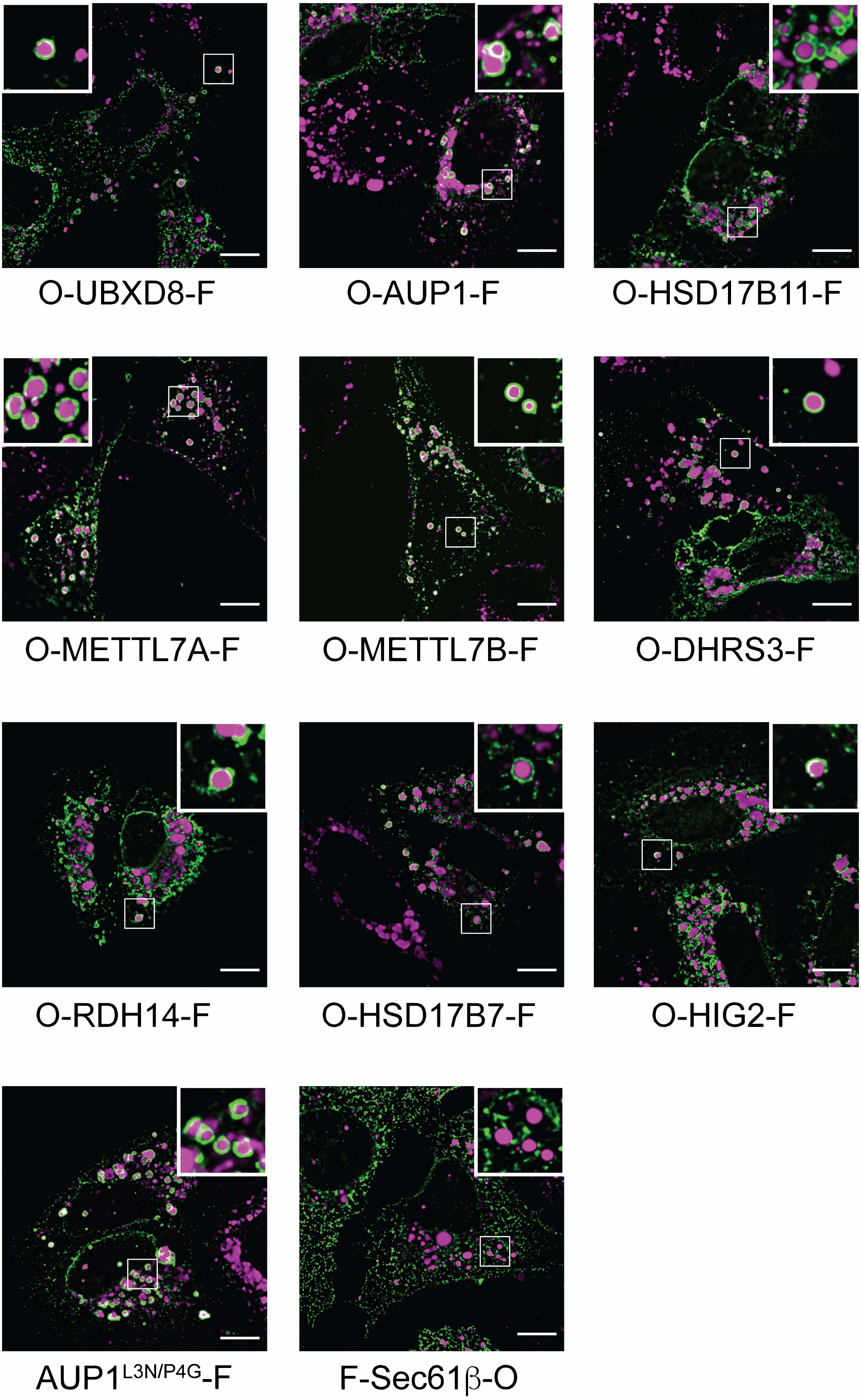
O-UBXD8-F
O-AUP1-F
O-HSD17B11-F
O-METTL7A-F
O-METTL7B-F
O-DHRS3-F
O-RDH14-F
O-HSD17B7-F
O-HIG2-F
AUP1$^{L3N/P4G}$-F
F-Sec61β-O

Fig. S3. OPG2-tagged LD membrane proteins reach LDs.

Indicated LD membrane proteins tagged with an N-terminal OPG2 tag ("O") and a C-terminal FLAG epitope ("F") as well as an AUP1-FLAG variant (AUP1$^{L3N/P4G}$) lacking the OPG2 tag were transiently expressed in U2OS cells grown on coverslips and loaded with oleic acid. Cells were fixed with paraformaldehyde and stained with anti-FLAG antibody (green) and LD-specific dye, LipidToxRed (magenta). FLAG-Sec61β-OPG2 was used as a control, ER-resident membrane protein. Wide-field microscopy images of a single Z-stack are shown. Scale bar – 10 μm.

Journal of Cell Science • Supplementary information

Figure S4

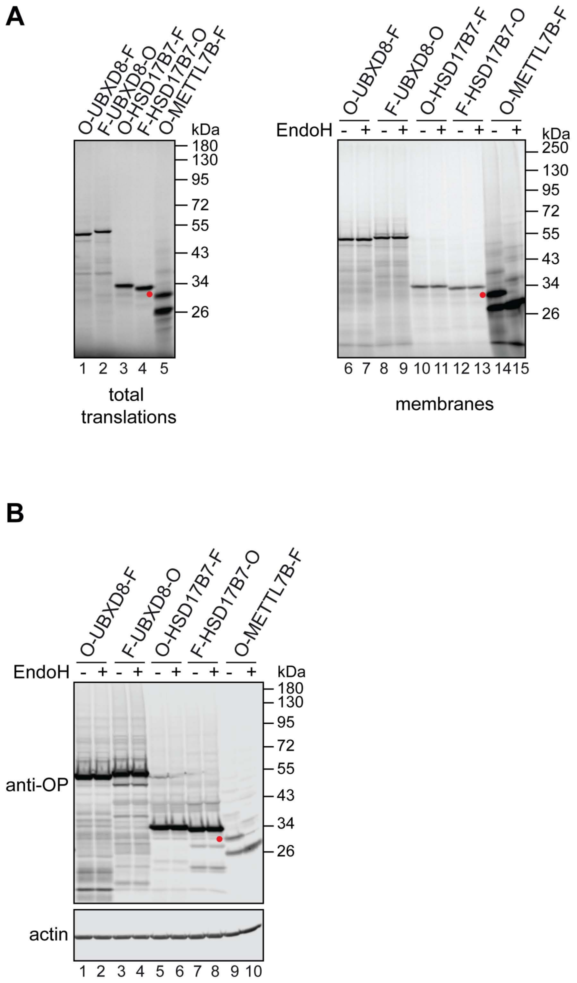

Fig. S4. Neither the N- nor the C-terminus of UBXD8 and HSD17B7 translocate to the ER lumen.

(A) UBXD8 and HSD17B7 tagged with the OPG2 epitope (“O”) either at the N- or C-terminus, and with the FLAG epitope (“F”) at the opposite end, were translated *in vitro* in the presence of ER-derived microsomes, membranes were isolated and, where indicated, treated with Endoglycosidase H (EndoH). Total translation reactions and the membrane fractions were resolved by SDS-PAGE and results were visualised by phosphorimaging. OPG2-METTL7B-FLAG was used as an N-glycosylated control protein (indicated with a red dot). **(B)** The same constructs as in (A) were transiently expressed in U2OS cells, which were then lysed and, where indicated, treated with EndoH. Samples were resolved by SDS-PAGE and results visualised by Western blotting with antibodies against rhodopsin (anti-OP) and actin.

Journal of Cell Science • Supplementary information

Figure S5

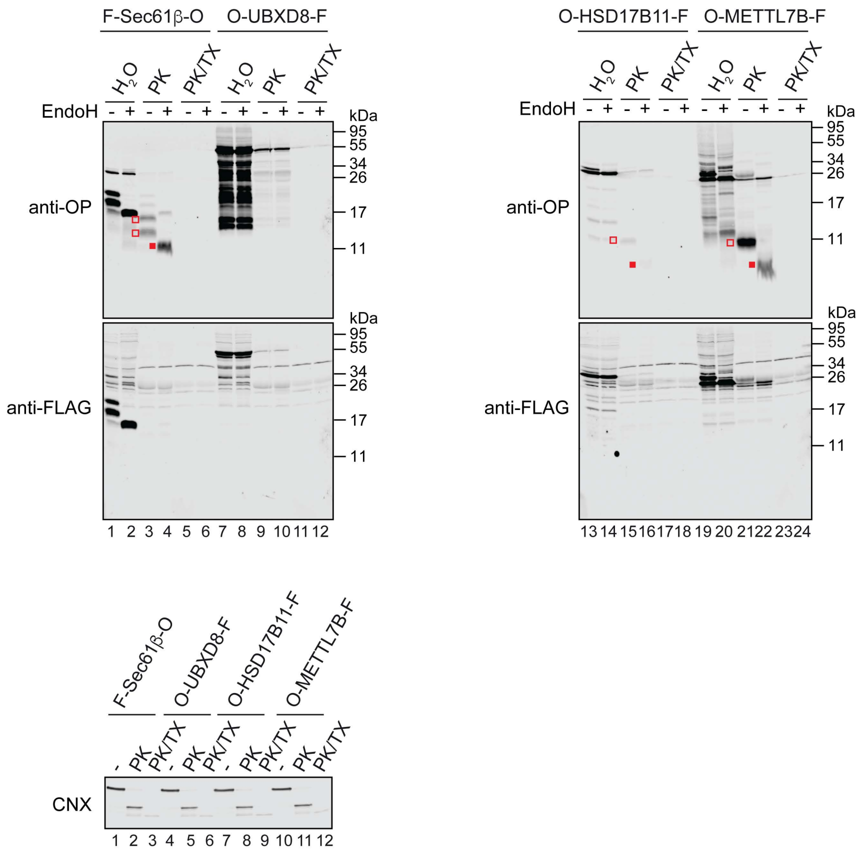

Fig. S5. N-glycosylation traps LD membrane proteins in a fully membrane-spanning topology.

Indicated membrane proteins tagged with the wild-type OPG2 (“O”) and the FLAG (“F”) epitopes were expressed in HeLa cells, the plasma membrane selectively permeabilised with digitonin and protein accessibility to proteinase K (PK) tested. Where indicated, reactions were supplemented with Triton X-100 (TX) to lyse intracellular membranes, including the ER. Samples were treated with EndoH or buffer control, resolved by SDS-PAGE and used for Western blotting with anti-rhodopsin (OP), anti-FLAG and anti-calnexin (CNX) antibodies. Open, red squares indicate N-glycosylated, protease-protected fragments whilst filled, red squares correspond to de-glycosylated, protease-protected species.

Journal of Cell Science • Supplementary information

Figure S6

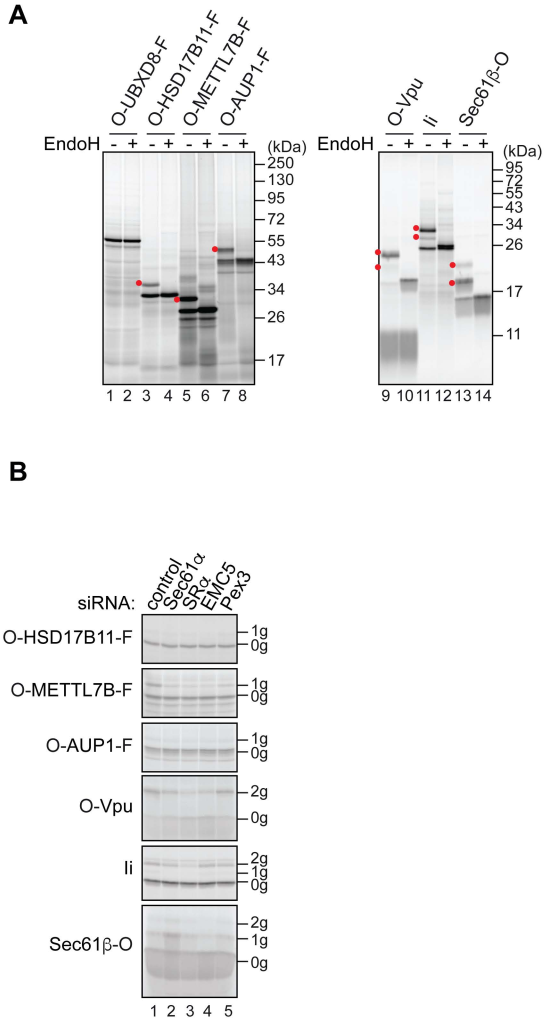

Fig. S6. Translation and N-glycosylation of LD membrane proteins in semi-permeabilised HeLa cells.

(A) HeLa cells were selectively permeabilised with digitonin and used as a source of ER membrane during *in vitro* translation of the indicated LD membrane proteins carrying an N-terminal OPG2 tag ("O") and a C-terminal FLAG epitope ("F"). N-terminally OPG2-tagged Vpu, C-terminally OPG2-tagged Sec61β and untagged invariant chain (Ii) were also used. The membrane fraction is shown which, where indicated, was treated with EndoH, resolved by SDS-PAGE and results visualised by phosphorimaging. Red dots indicate N-glycosylated protein species. **(B)** Aliquots of the total translation products from reactions used in Fig. 6B of the main text are shown where "0g" indicates non-glycosylated, "1g" singly N-glycosylated and "2g" doubly N-glycosylated protein species.

Table S1. Efficiency of membrane protein knock-downs.

Indicated membrane proteins implicated in protein biogenesis at the ER were depleted in HeLa cells via siRNA-mediated knock-down as shown in Fig. 6A of the main text. Signals obtained by quantitative Western blotting were normalised to lamin B, which was used as a loading control, and expressed as a ratio to control siRNA-treated cells. Mean depletion and standard error of mean (SEM) are shown for n=4 biological replicates.

Table S1

	Sec61α	SRα	EMC5	Pex3
knock-down efficiency (%)	84.92	96.40	90.14	90.60
SEM	0.65	0.42	7.44	0.90

Journal of Cell Science • Supplementary information
