# Supplementary material for: Co-translational biogenesis of lipid droplet integral membrane proteins
Source: J Cell Sci. 2021 Nov 2;135(5):jcs259220. doi: 10.1242/jcs.259220 (PMC8627552; doi:10.1242/jcs.259220)
Supplement: Reviewer comments [file joces-135-259220_review_history.pdf]

Journal of Cell Science

Co-translational biogenesis of lipid droplet integral membrane proteins

Pawel Leznicki, Hayden O. Schneider, Jada V. Harvey, Wei Q. Shi and Stephen High
DOI: 10.1242/jcs.259220

Editor: James Olzmann

Review timeline

Original submission:	5 August 2021
Editorial decision:	8 September 2021
First revision received:	16 September 2021
Accepted:	16 September 2021

Original submission

First decision letter

MS ID#: JOCES/2021/259220

MS TITLE: Co-translational biogenesis of lipid droplet integral membrane proteins

AUTHORS: Pawel Leznicki, Hayden O Schneider, Jada V Harvey, Wei Q Shi, and Stephen High
ARTICLE TYPE: Research Article

We have now reached a decision on the above manuscript.

To see the reviewers' reports and a copy of this decision letter, please go to: https://submit-jcs.biologists.organd click on the 'Manuscripts with Decisions' queue in the Author Area. (Corresponding author only has access to reviews.)

As you will see, the reviewers gave favorable reports but raised some critical points that will require amendments to your manuscript. Specifically, there are some concerns raised by reviewer #2 related to the conclusion that the N-terminus of LD proteins accesses the ER lumen. I hope that you will be able to address these comments and I look forward to seeing a revised version of this very interesting work.

We are aware that you may be experiencing disruption to the normal running of your lab that makes experimental revisions challenging. If it would be helpful, we encourage you to contact us to discuss your revision in greater detail. Please send us a point-by-point response indicating where you are able to address concerns raised (either experimentally or by changes to the text) and where you will not be able to do so within the normal timeframe of a revision. We will then provide further guidance. Please also note that we are happy to extend revision timeframes as necessary.

Please ensure that you clearly highlight all changes made in the revised manuscript. Please avoid using 'Tracked changes' in Word files as these are lost in PDF conversion.

I should be grateful if you would also provide a point-by-point response detailing how you have dealt with the points raised by the reviewers in the 'Response to Reviewers' box. Please attend to all of the reviewers' comments. If you do not agree with any of their criticisms or suggestions please explain clearly why this is so.

© 2021. Published by The Company of Biologists under the terms of the Creative Commons Attribution License (https://creativecommons.org/licenses/by/4.0/).

Reviewer 1

Advance summary and potential significance to field

The findings presented by Leznicki et al. represent several advances in the field of lipid droplet (LD) protein biology.

The authors meticulously characterised the requirements of LD-resident protein biogenesis and show that LD protein membrane insertion can occur either co- or posttranslationally (Fig 1). Intriguingly, most LD proteins temporarily gain access to the ER lumen as the authors have compellingly demonstrated using glycosylation tag-based assays (Figs 2 + 3). They next demonstrated that the co- vs posttranslational route of membrane insertion is determined by the transmembrane region (Fig 4) and that insertion of LD proteins was independent of the Sec61 insertase activity (Fig 5). Finally, this work identifies the EMC as a biogenesis factor for LD proteins – potentially collaborating with SR and 'non-insertase activities' of Sec61 (Fig 6).

This study advances our knowledge of LD protein biogenesis and, more specifically, reveals an involvement of the EMC in their correct membrane insertion.

Comments for the author

I have no major concerns relating to this carefully conducted study.
Minor questions that might be addressed by the authors are listed below.

Minor technical comments
Figure 1B left: The membrane and total translation radiographs seem slightly misaligned and realignment would facilitate readability of the figure.
Figure 3C: The authors write that none of the LD proteins showed PK protection, but one could interpret some of the lower molecular weight bands in lane 8 as potentially PK protected? However, this can be particular to O*-HSD17B11-F as it is among a subset of LD proteins that are efficiently glycosylated (Fig 2B). I would suggest rephrasing the statement slightly.

Minor conceptual comments
It is clear from the data presented here that the EMC is involved in LD protein biogenesis. Some open questions have been addressed in the discussion. The points raised in the comments below go beyond what is necessary for publication and should therefore not be considered essential to be addressed experimentally in this manuscript.

It would be nice to see whether the LD proteins under investigation bind to the EMC. Even though not absolutely necessary, this could be addressed using co-IP experiments. Alternativley, searching publicly available proteomics data sets from co-IP experiments of individual EMC subunits (i.e. Tian et al, DOI:10.1016/j.celrep.2019.08.006; Shurtleff et al, DOI: 10.7554/eLife.37018) might reveal LD proteins binding to the EMC, further substantiating the experimental data shown in this work. However, detection of clients binding the EMC might be challenging due to the transient nature of their interaction.

The authors have shown that abrogating the EMC by knocking down one of its core subunits, EMC5, reduces the glycosylation of most of the tested LD proteins. It would be interesting whether in intact cells, these proteins now aggregate, are targeted for degradation or are they mistargeted to other subcellular membrane compartments? If data on some of these questions should already be available, it might be nice to include or shortly discuss them in the manuscript. However, I consider these points wholly optional in the context of this work and do not expect the authors to perform additional experiments.

Another question pertaining to the biochemistry of the EMC is beyond the confines of this manuscript. The EMC consists of 10 subunits in mammalian cells and it is currently not entirely clear whether some of the subunits might modulate some aspects of EMC specificity etc. In this regard, it might be interesting to test whether some EMC subunits (whose depletion does not affect EMC abundance (i.e. EMC4, EMC10)) are dispensible for correct LD protein biogenesis. This would shed more light on the various reported functions of the EMC as insertase and chaperone for

© 2021. Published by The Company of Biologists under the terms of the Creative Commons Attribution License (https://creativecommons.org/licenses/by/4.0/).

polytopic membrane proteins. While this might be a worthwhile exercise, in the context of this work, I consider any additional experiments addressing this question as fully optional and not critical to the publication of the findings presented in the manuscipt.

Overall, I have no major concerns and think this work is a significant and novel contribution in the fields of LD protein biogenesis and EMC biology. The data presented justify the conclusions drawn. Furthermore, the subject matter and findings are of broad interest and warrant publication in JOCES. I would be happy if the authors could address the ‘minor technical comments’, but I do not expect the authors to experimentally address the ‘minor conceptual comments’ above.

Reviewer 2

Advance summary and potential significance to field

The manuscript by Leznicki et al. investigates how lipid droplet (LD) membrane proteins integrate into the ER membrane prior to trafficking to LDs. Using in vitro assays, the authors show that ER membranes must be present during the synthesis of LD membrane proteins for membrane integration through a mechanism dependent on the ER membrane protein complex (EMC). The data supporting these claims are strong and will be of wide interest to the field. The authors additionally use N-linked glycosylation motifs to suggest that the N-terminus of certain LD membrane proteins transiently access the ER lumen before assuming the hairpin topology needed to reside in the monolayer of LDs. However, this mechanistic model primarily relies on correlational observations that do not sufficiently address the order of events. The manuscript would be more accurate if more direct assays were included, or if the claims related to these observations were toned down.

Comments for the author

The main concern relates to suggestion that the N-terminus of LD membrane proteins are transiently exposed to the ER lumen before rearrangement into a hairpin topology (Fig. 2-3). As the authors state, most of the presented data cannot rule out the possibility that glycosylated portion of proteins that have gained access to ER lumen are a distinct population from those that ultimately get trafficked to LDs (pg. 10), and glycosylation may independently prevent topological rearrangement (pg. 11). More direct assays should be included to support Fig. 3A, which is the only result that attempts to simultaneously assay the population of LD-trafficked proteins that have been transiently exposed to the ER lumen. Alternatively, less emphasis should be placed on this model in the abstract and throughout the manuscript.

1. The authors claim that the "1g" band in Fig. 3A is EndoH-sensitive. This result--specifically where the fractionation is performed prior to EndoH treatment--should be included.

2. The authors use Fig. 3A to suggest that at least some glycosylated segments can still assume the hairpin topology and reach LDs. Additionally showing that the glycan is accessible to cytosolic activities biochemically or by microscopy (e.g. adding a glycanase in the absence of detergent following fractionation or by using a labeled lectin-binding partner) would more directly support this conclusion.

3. Limitations such as the influence of glycosylation on topology may indicate that glycosylation may not be the best (or at least should not be the only) readout used to build this mechanistic model. Other compartment-specific activities, including localized sequence-specific proteases or biotinylation, are established alternative options to possibly probe that the topological rearrangement occurs as the authors suggest.

© 2021. Published by The Company of Biologists under the terms of the Creative Commons Attribution License (https://creativecommons.org/licenses/by/4.0/).

First revision

Author response to reviewers' comments

Point-by-point response to reviewers' comments:

We are grateful to both reviewers for their constructive comments and suggestions. We provide a point-by-point response below. Please note that for clarity we have identified the relevant textual changes in our revised manuscript using yellow highlights.

Reviewer 1 Comments for the Author:

We thank Reviewer 1 for their conclusion that our work "advances our knowledge of LD protein biogenesis and, more specifically, reveals an involvement of the EMC in their correct membrane insertion" and concluding that they "have no major concerns relating to this carefully conducted study"

Minor technical comments:
Point 1. Figure 1B left: The membrane and total translation radiographs seem slightly misaligned and realignment would facilitate readability of the figure.

Response: This alignment issue was primarily due to the "smiling" of the samples in the membrane fraction panel towards the bottom of the original gel. For clarity we have now both labelled and numbered the individual lanes in both the upper and lower panels of this Fig. 1B (see new version of Figure 1 provided).

Point 2. Figure 3C: The authors write that none of the LD proteins showed PK protection, but one could interpret some of the lower molecular weight bands in lane 8 as potentially PK protected? However, this can be particular to O*-HSD17B11-F as it is among a subset of LD proteins that are efficiently glycosylated (Fig 2B). I would suggest rephrasing the statement slightly.

Response: In the data presented in Figure 3C, we do not see any PK protected fragments that are equivalent to those observed in the "glycan-trapping" experiments provided in Supplementary Figure S5. Here we can see discrete EndoH sensitive PK protected fragments bearing the OPG tag that must be derived from the N-termini of O-HSD17B11-F and O- METTL7B-F. Our original text concluding that there are no "comparable membrane protected fragments" related to this observation. However, as Reviewer 1 points out, some larger potential PK protected fragments are apparent in lane 8. We have now indicated the relevant species using filled black circles (see Fig. 3C) and modified the text so that it now reads "comparable membrane protected fragments were not apparent with the LD membrane proteins tested". We have also added the following sentence to the end of the paragraph where we address these species: For O*-HSD17B11-F, some larger PK protected fragments are faintly visible (Fig. 3C, cf. lanes 7 and 8, filled black circles, "anti-OP" panel), suggesting that a fraction of the newly synthesised protein may not have fully re-oriented.

Response to minor conceptual comments:
We thank Reviewer 1 for their thoughtful suggestions, some of which were on our "to do list" with the others now added. In line with the comments provided by Reviewer 2, we have initially rewritten our resubmitted manuscript to place less emphasis on our model for LD membrane protein topological rearrangements. We hope to address such further conceptual questions (Reviewer 1) and employ additional technical approaches (Reviewer 2) in our follow up work.

Reviewer 2 Comments for the Author:

Major point: The main concern relates to suggestion that the N-terminus of LD membrane proteins are transiently exposed to the ER lumen before rearrangement into a hairpin topology (Fig. 2-3). As the authors state, most of the presented data cannot rule out the possibility that glycosylated portion of proteins that have gained access to ER lumen are a distinct population

© 2021. Published by The Company of Biologists under the terms of the Creative Commons Attribution License (https://creativecommons.org/licenses/by/4.0/).

from those that ultimately get trafficked to LDs (pg. 10), and glycosylation may independently prevent topological rearrangement (pg. 11). More direct assays should be included to support Fig. 3A, which is the only result that attempts to simultaneously assay the population of LD-trafficked proteins that have been transiently exposed to the ER lumen. **Alternatively, less emphasis should be placed on this model in the abstract and throughout the manuscript**.

Response: We appreciate the thoughtful comments of reviewer 2 and their suggestion that we could **place less emphasis on our model for the topological rearrangement** of newly synthesised LD membrane proteins as an alternative to carrying out extensive additional experimentation. To this end we have revised our text as follows with yellow highlights indicating the relevant changes and modifications:

Summary statement now reads: Insertion of many lipid droplet membrane proteins into the endoplasmic reticulum (ER) is co-translational, mediated by the ER membrane protein complex (EMC) and may involve topology reorientation.

Abstract now reads: This route may even result in a transient exposure of the short N-termini of some LD membrane proteins to the ER lumen, followed by putative topological rearrangements that would enable their transmembrane segment to form a hairpin loop and N-termini to face the cytosol.

Results section now contains the following newly added text: However, whilst the co-fractionation of N-glycosylated OPG2-HSD17B11-FLAG with LDs (Figs. 3A and 3B) supports this model, we cannot rule out the alternative possibility that two topologically distinct populations of LD membrane proteins are synthesised, and that these two cohorts have distinct fates (see Discussion).

Discussion section now contains the following newly added text: It should be noted that on the basis of our current data we cannot formally exclude the possibility that two distinct populations of LD proteins are synthesised via this co-translational route. One population that immediately assumes a hairpin conformation on the cytosolic leaflet of the ER membrane and subsequently enters LDs and a second that spans the membrane with its N-terminus in the ER lumen but is unable to acquire a hairpin topology. In the latter case, such proteins may be recognised as aberrant or mis-inserted resulting in their ER associated degradation (Wu and Rapoport 2018).

Legend to Figure 7: We speculate that the co-translationally delivered LD membrane proteins transiently expose their N-terminus to the ER lumen but then re-arrange their topology to form a hairpin with both termini facing the cytosol, which is a prerequisite for trafficking to LDs. Such topological re-orientation could occur either co-translationally (route 2a) or following complete protein synthesis (route 2b).

In addition the following changes have been made to the Discussion:

Change 1: At present our favoured model for the EMC-mediated co-translational biogenesis of LD proteins incorporates the possibility that newly synthesised polypeptides may reorient from a fully membrane-spanning topology to a hairpin one, in order to be accommodated by LDs (cf. (Abell et al., 2002; Stevanovic and Thiele, 2013; Zehmer et al., 2009; Zehmer et al., 2008)).
The normally transient nature of such N-terminal domain residency in the ER lumen is supported by our protease protection studies.

Change 2: At present we can only speculate as to how the re-orientation of such LD membrane proteins might occur.

Change 3: How LD membrane proteins might transition from a fully membrane-spanning topology to a hairpin one and whether this process is spontaneous or requires dedicated factors are key questions for future studies.

Change 4: The majority of a paragraph where we speculated about the possible contribution of the TMD region of an LD membrane protein to its hypothetical re-orientation, which originally followed the statement that “the ER-LD interface appears to act as a barrier that can exclude

© 2021. Published by The Company of Biologists under the terms of the Creative Commons Attribution License (https://creativecommons.org/licenses/by/4.0/).

fully membrane-spanning proteins from LDs (Khaddaj et al., 2022)” has now been removed from our revised discussion.

Specific points:

Point 1. The authors claim that the "1g" band in Fig. 3A is EndoH-sensitive. This result--specifically where the fractionation is performed prior to EndoH treatment--should be included.

Response: We now include our data confirming that the “1g” species shown in the LD fraction of Fig. 3A is EndoH sensitive as an additional inset panel in Fig. 3A (see new version of Figure 3 provided) and the legend to Figure 3 has been updated accordingly.

Point 2. The authors use Fig. 3A to suggest that at least some glycosylated segments can still assume the hairpin topology and reach LDs. Additionally showing that the glycan is accessible to cytosolic activities biochemically or by microscopy (e.g. adding a glycanase in the absence of detergent following fractionation or by using a labeled lectin-binding partner) would more directly support this conclusion.

Point 3. Limitations such as the influence of glycosylation on topology may indicate that glycosylation may not be the best (or at least should not be the only) readout used to build this mechanistic model. Other compartment-specific activities, including localized sequence- specific proteases or biotinylation, are established alternative options to possibly probe that the topological rearrangement occurs as the authors suggest.

Response to Points 2 and 3: Having rewritten our resubmitted manuscript to place less emphasis on our model for LD membrane protein topological rearrangements, we hope to include techniques such as these in our follow up work

---

Second decision letter

MS ID#: JOCES/2021/259220

MS TITLE: Co-translational biogenesis of lipid droplet integral membrane proteins

AUTHORS: Pawel Leznicki, Hayden O Schneider, Jada V Harvey, Wei Q Shi, and Stephen High
ARTICLE TYPE: Research Article

I am happy to tell you that your manuscript has been accepted for publication in Journal of Cell Science, pending standard ethics checks. Thank you for submitting this exciting work to J Cell Science!

© 2021. Published by The Company of Biologists under the terms of the Creative Commons Attribution License (https://creativecommons.org/licenses/by/4.0/).
